# Multi-Strategy Improved Pelican Optimization Algorithm for Engineering Optimization Problems and 3D UAV Path Planning

**DOI:** 10.3390/biomimetics11010073

**Published:** 2026-01-15

**Authors:** Ming Zhang, Maomao Luo, Huiming Kang

**Affiliations:** 1 Alibaba Business School, Hangzhou Normal University, Hangzhou 311121, China; 20210030@hznu.edu.cn; 2 Public Education Department, Jiujiang Polytechnic University of Science and Technology, Jiujiang 332020, China; jzmm1991@outlook.com; 3 College of Economics and Management, Shenyang Aerospace University, Shenyang 110136, China; 4 School of Information Technology, Monash University, Malaysia Campus, Bandar Sunway 47500, Selan, Malaysia

**Keywords:** pelican optimization algorithm, metaheuristic algorithm, path planning, engineering optimization

## Abstract

To address the path-planning challenge for unmanned aerial vehicles (UAVs) in complex environments, this study presents an improved pelican optimization algorithm enhanced with multiple strategies (MIPOA). The proposed method introduces four main improvements: (1) using chaotic mapping to spread the initial search points more evenly, thereby increasing population variety; (2) incorporating a random Lévy-flight strategy to improve the exploration of the search space; (3) integrating a differential evolution approach based on Cauchy mutation to strengthen individual diversity and overall optimization ability; and (4) adopting an adaptive disturbance factor to speed up convergence and fine-tune solutions. To evaluate MIPOA, comparative tests were carried out against classical and modern intelligent algorithms using the CEC2017 and CEC2022 benchmark sets, along with a custom UAV environmental model. Results show that MIPOA converges faster and achieves more accurate solutions than the original pelican optimization algorithm (POA). On CEC2017 in 30-, 50-, and 100-dimensional cases, MIPOA attained the best average ranks of 1.57, 2.37, and 2.90, respectively, and achieved the top results on 26, 21, and 19 test functions, outperforming both POA and other advanced algorithms. For CEC2022 (20 dimensions), MIPOA obtained the highest Friedman average rank of 1.42, demonstrating its effectiveness in complex UAV path-planning tasks. The method enables the generation of faster, shorter, safer, and collision-free flight paths for UAVs, underscoring the robustness and wide applicability of MIPOA in real-world UAV path-planning scenarios.

## 1. Introduction

In recent years, the swift expansion of general aviation and the unmanned aerial vehicle (UAV) industry has catalyzed the rise in the “low-altitude economy” [1]. This emerging economic paradigm encompasses diverse low-altitude flight operations involving both manned and unmanned aircraft, fostering integration and development across numerous related sectors. Representative platforms within this domain include UAVs, electric vertical takeoff and landing (eVTOL) aircraft, helicopters, and conventional fixed-wing airplanes, with applications extending to residential, industrial, and commercial settings.

Among these platforms, UAVs have seen widespread deployment in fields such as logistics, aerial photography, military missions, public safety, traffic monitoring, and remote sensing [2,3,4]. Central to UAV operations is path planning, which refers to the procedure of identifying an optimal or near-optimal trajectory from a starting point to a designated destination [5,6,7]. Effective path planning is essential for ensuring mission accomplishment and operational safety [8,9]. Indeed, a well-designed route that balances safety, feasibility, computational efficiency, and cost significantly improves the overall effectiveness of UAV missions.

The requirements for path planning vary with mission type. For tasks such as aerial photography, mapping, and surface inspection, the flight path should be minimized in length to reduce time and energy expenditure [10,11,12,13]. In contrast, dynamic missions like target search, surveillance, or rescue operations require paths optimized according to criteria such as maximizing detection likelihood or minimizing flight duration. Furthermore, environmental safety and UAV performance constraints—including collision avoidance, flight time, altitude limits, and fuel consumption—must be carefully considered [14,15,16]. Consequently, generating collision-free and feasible trajectories through efficient path planning remains a challenging endeavor, often necessitating dedicated optimization algorithms to attain satisfactory results. Although this work focuses on single-UAV path planning, recent studies by M. Irani Rahaghi et al. [17,18] offer valuable insights: cooperative UAV-unmanned ground vehicle (UGV) operations and nonlinear optimal path-tracking for robotic manipulators represent promising directions for future intelligent transportation and autonomous systems, with expected impacts in logistics, emergency services, and related fields.

To address such planning challenges, optimization methods are indispensable. Optimization is the process of identifying the best solution that satisfies specified objectives, conditions, or criteria [17,18,19]. Typically, an optimization problem admits multiple candidate solutions [20], and the aim is to select the most suitable one from the available set [21]. Optimization approaches are broadly divided into two categories: deterministic and stochastic methods [22]. The choice depends on the problem’s characteristics, available information, scale, and required precision. Deterministic methods conduct a systematic search for the optimum, whereas stochastic techniques are generally employed for complex, multimodal problems with numerous local optima. Although stochastic methods can escape local traps, they often demand greater computational resources and time. Classical deterministic approaches (e.g., A*) exhibit high computational complexity and poor adaptability in three-dimensional path planning; as a result, meta-heuristic algorithms, which mimic natural phenomena and balance global exploration with local exploitation, have consequently gained prominence. As conventional planning methods struggle in 3D environments, researchers have increasingly turned to heuristic techniques, especially swarm intelligence (SI) algorithms [23,24,25].

Despite the growing adoption of UAVs in logistics, emergency response, military reconnaissance, and other domains, path planning under complex terrain, dynamic obstacles, and multiple constraints remains a significant challenge. Traditional methods often prove inefficient in 3D spaces and are prone to becoming trapped in local optima. While swarm-intelligence algorithms offer certain advantages, they still suffer from drawbacks such as slow convergence, inadequate population diversity, and premature convergence. Therefore, this paper aims to develop an efficient, stable, and highly adaptable path-planning method to enhance UAV mission performance in complex environments. To tackle these optimization challenges, a wide array of stochastic optimization algorithms have been devised. Based on their underlying inspiration, they can be grouped into five primary categories: evolution-based, physics-based, game-based, human-based, and swarm-intelligence-based algorithms [26,27,28,29].

Among these, swarm intelligence algorithms have been particularly active in recent years. For example, the Gravitational Search Algorithm (GSA) [30] emulates gravitational interactions among masses to locate optimal solutions. The Gray Wolf Optimizer (GWO) [31] draws inspiration from the social hierarchy and cooperative hunting of gray wolf packs. The Moth-Flame Optimization (MFO) algorithm [32] models the transverse orientation navigation of moths. The Whale Optimization Algorithm (WOA) [33] simulates the social behavior of whale pods, while the Snake Optimizer (SO) [34] is inspired by the mating rituals of snakes. The Crow Search Algorithm (CSA) [35] replicates the intelligent food-searching behavior of crows.

More recently, several new swarm-intelligence algorithms have emerged. The African Vulture Optimization Algorithm (AVOA) [36] mimics the foraging and navigation tactics of African vultures, whereas the Dung Beetle Optimizer (DBO) [37] simulates various dung-beetle behaviors, including rolling, foraging, and reproduction. The Aquila Optimizer (AO) [38] is grounded in the hunting strategies of eagles, and the Harris Hawks Optimization (HHO) [39] replicates the cooperative “surprise pounce” hunting technique of Harris’s hawks. A hybrid approach combining particle swarm optimization and gravitational search with chaotic maps (CPSOGSA) [40] has also been proposed to improve exploration and exploitation capabilities.

One notable recent contribution is the Pelican Optimization Algorithm (POA) [41], introduced by Trojovský et al., a meta-heuristic algorithm inspired by the foraging behavior of pelicans. POA has shown commendable performance on benchmark functions, surpassing eight typical swarm-intelligence algorithms. It offers favorable convergence accuracy, rapid optimization speed, and a low parameter count, rendering it user-friendly and broadly applicable. However, POA is hampered by issues such as insufficient population diversity and a tendency to converge prematurely to local optima. Like other heuristic algorithms, its performance can vary with the problem at hand; for instance, it is prone to local stagnation in tasks such as slope-stability prediction. Li et al. [42] developed an intelligent slope-stability prediction method based on an improved pelican optimization algorithm (IPOA) and an optimized random forest (RF) algorithm to mitigate hazards caused by slope instability. Qiao et al. [43] observed that POA also suffers from imbalanced global exploration and a propensity for local optima; to accurately predict reservoir porosity, they proposed a bidirectional long short-term memory model with an attention mechanism (BiLSTM-AM) optimized by an improved pelican optimization algorithm (IPOA). Seyed-Garmroudi et al. [44] further noted that although POA converges quickly, it exhibits premature convergence, an imbalance between exploration and exploitation, and a lack of population diversity. Their work introduced an improved POA (IPOA) to alleviate these shortcomings.

To address the aforementioned limitations of POA, this study presents a Multi-strategy Improved Pelican Optimization Algorithm (MIPOA). The principal contributions of this paper are as follows:

(1) Multi strategy Improved Pelican Optimization Algorithm: To overcome POA’s drawbacks, including its susceptibility to local optima and limited accuracy, MIPOA integrates four enhancement strategies into the original algorithmic framework. These modifications render the search process more systematic and continuous, thereby improving population diversity, global search capability, and overall optimization performance.
(2) Application to UAV Path Planning: MIPOA is successfully employed for UAV path planning tasks. Competitive comparisons with other heuristic algorithms demonstrate its superior performance and adaptability across a variety of path planning challenges.
(3) Paper Organization: The remainder of this paper is structured as follows. Section 2 formulates the UAV mathematical model. Section 3 briefly outlines the principles of the original POA. Section 4 elaborates on the four strategy improvements incorporated in MIPOA. Section 5 evaluates MIPOA’s performance through comparative experiments and scenario specific analyses. Section 6 applies MIPOA to simulated UAV path planning experiments. Finally, Section 7 concludes the study and suggests directions for future work.

## 2. UAV Mathematical Model

The trajectory of an unmanned aerial vehicle (UAV) during operation is predominantly shaped by environmental and regulatory factors, including terrain features, flight altitude, meteorological conditions, and restricted no-fly zones [45]. To safeguard both the vehicle and its operational integrity, flight paths are typically designed to circumvent hazardous regions such as mountainous areas, confined passages, and prohibited airspaces. Consequently, a path-planning algorithm must determine an optimal or near-optimal route that safely and efficiently connects the designated origin and destination points [46,47,48].

Assuming the UAV maintains a constant cruise speed, the path-planning task can be simplified to a static poly-line optimization problem. In light of typical UAV operational requirements, a comprehensive cost function is formulated by aggregating four principal components: travel distance, flight altitude, threat exposure, and trajectory smoothness. Mathematically, the total cost is expressed as:

(1) $$F\left(X_{i}\right)=\sum_{c=1}^{4}\;F_{c}\left(X_{i}\right)=\sum_{c=1}^{4}\;b_{c}F_{c}(X_{i})$$

where $b_{c}$ denote weighting coefficients, with $b_{1}=b_{2}=1,b_{3}=10\;and\;b_{4}=1$. The terms $F_{1}\left(X_{i}\right)$ through $F_{4}\left(X_{i}\right)$ correspond to the path-length cost, threat cost, altitude cost, and smoothness cost, respectively. The decision variable $X_{i}$ comprises an ordered sequence of l waypoints $P_{ij}=(x_{ij},y_{ij},z_{ij})$, each satisfying $P_{ij}\in O$, with O representing the UAV’s feasible operational space.

### 2.1. Path Length Cost

Flight distance directly influences mission efficiency, duration, and operational expenditure. To achieve timely and economical operations while preserving safety, a primary objective is to minimize the total path length. The corresponding cost $F_{1}$ is defined as the cumulative Euclidean distance between consecutive waypoints, computed as:

(2) $$F_{1}\left(X_{i}\right)=\sum_{j=1}^{l-1}\;\left\|\vec{P_{ij}P_{i,j+1}}\right\|.$$

here, $P_{ij}$ and $P_{i,j+1}$ denote two adjacent nodes in the path. The total length cost thus equals the sum of the l−1 line segments formed by the l waypoints.

### 2.2. Safety and Feasibility Costs

Beyond minimizing path length, UAV path planning must also ensure operational safety. This involves avoiding designated threat zones, such as no-fly areas (e.g., populated regions or zones affected by adverse weather) [49]. Owing to the complexity of threat modeling and the challenges in acquiring real-world data, this work abstracts the threat environment by representing each threat zone as a cylinder of fixed radius. The effective radius of the threat zone corresponds to the cylinder’s radius. Defining such threat zones aims to create a viable flight environment, enabling the UAV to safely circumvent hazardous areas and accomplish its mission.

**Figure 1:**
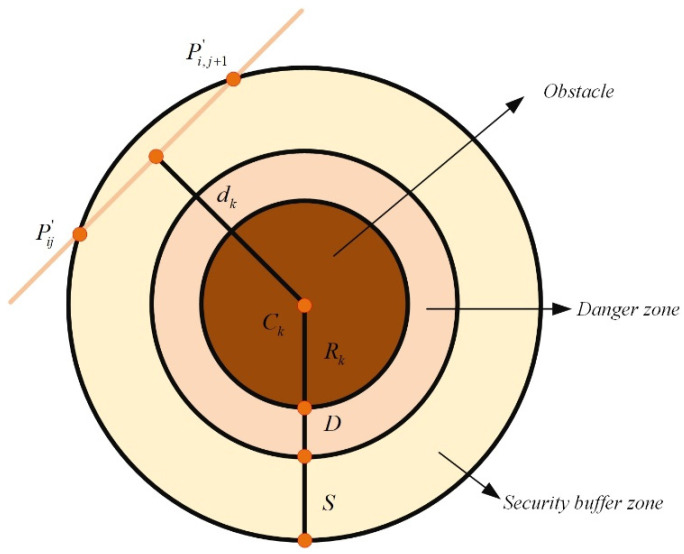
Safety constraint cost.

Let K denote the set of all threats, with each threat confined within a cylindrical region. The cylinder’s projection center is denoted as $C_{k}$, and its radius is $R_{k}$. Figure 1 illustrates the safety-constraint configuration. During flight, the UAV must also steer clear of tall ground-based obstacles. The safety and feasibility cost $F_{2}$ is evaluated using Equations (3) and (4).

(3) $$F_{2}(X_{i})=\sum_{j=1}^{l-1}\;\sum_{k=1}^{K}\;T_{k}(\vec{P_{ij}P_{i,j+1}})$$

(4) $$T_{k}(\vec{P_{ij}P_{i,j+1}})=\left\{\begin{array}{cc} 0, & \mathrm{if}\;d_{k}>S+D+R_{k},\\ \left(S+D+R_{k}\right)-d_{k}, & \mathrm{if}\;D+R_{k}<d_{k}\leq S+D+R_{k},\\ \infty , & \mathrm{if}\;d_{k}\leq D+R_{k}. \end{array}\right.$$

where $d_{k}$ is the perpendicular distance from the path segment $\vec{P_{ij}P_{i,j+1}}$ to the threat center $C_{k}$, D stands for the UAV’s physical diameter, and S is an additional safety margin.

According to Equation (4), when $d_{k}$ falls within the danger zone ( $d_{k}\leq D+R_{k}$), the cost $F_{2}$ becomes infinite, forcing the UAV to remain far from the threat. If $d_{k}$ lies within the safety buffer ( $D+R_{k}<d_{k}\leq S+D+R_{k}$), the cost increases as the path approaches the obstacle. Once $d_{k}$ exceeds $S+D+R_{k}$, the safety cost reduces to zero, indicating that the path is sufficiently distant from the threat.

### 2.3. Flight Altitude Cost

**Figure 2:**
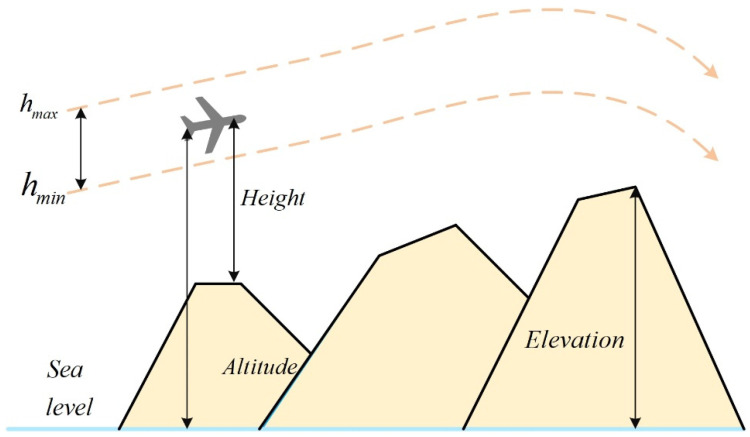
Highly constrained cost.

Flight altitude constitutes another critical operational constraint for UAVs. Depending on mission requirements, terrain features, obstacle distributions, airspace regulations, and task characteristics, permissible altitude ranges may vary. Figure 2 depicts the altitude-cost model. The UAV is required to operate between a minimum altitude $h_{min}$ and a maximum altitude $h_{max}$. The altitude penalty associated with waypoint $P_{ij}$ is computed as:

(5) $$H_{ij}=\left\{\begin{array}{cc} |h_{ij}-\frac{\left(h_{max}+h_{min}\right)}{2}|, & if{\;h}_{min}\leq h_{ij}\leq h_{max},\\ \infty , & otherwise. \end{array}\right.$$

where $h_{ij}$ denotes the altitude of waypoint $P_{ij}$ above ground level. The function $H_{ij}$ encourages flight near the mid-altitude of the permitted range and imposes an infinite penalty for violations. The total altitude cost $F_{3}$ is the summation of $H_{ij}$ over all waypoints:

(6) $$F_{3}\left(X_{i}\right)=\sum_{j=1}^{l}\;H_{ij}$$

### 2.4. Path Smoothing Cost

**Figure 3:**
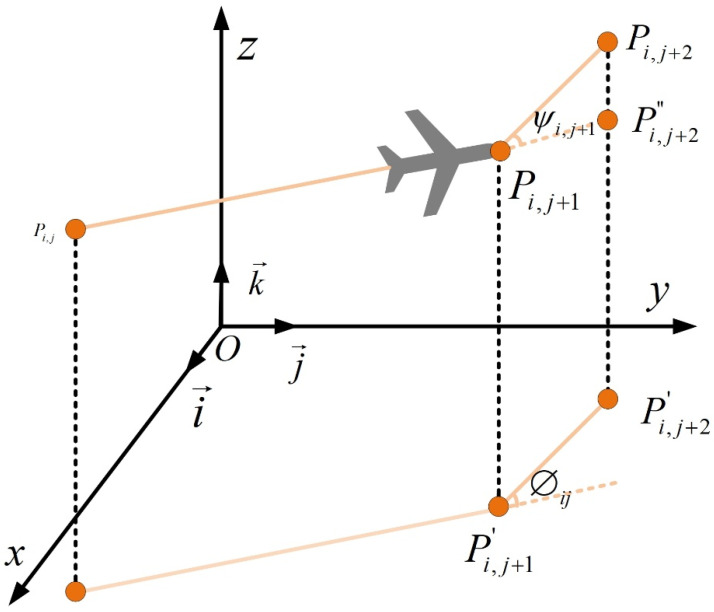
Turning angle and climbing angle constraints.

UAV maneuverability is largely governed by horizontal yaw and vertical pitch angles. To guarantee stable and safe flight, these angles must adhere to the vehicle’s physical limits; otherwise, the planned trajectory may become infeasible. Thus, constraints on the maximum turning angle are essential. Figure 3 illustrates the geometric relations for turning and climbing angles.

The turning angle $\phi_{ij}$ is defined as the angle between two consecutive path segments projected onto the horizontal plane: $\vec{P_{ij}^{\prime }P_{i,j+1}^{\prime }}$ and $\vec{P_{i,j+1}^{\prime }P_{i,j+2}^{\prime }}$. Let $\vec{k}$ be the unit vector along the vertical z-axis. The horizontal projection of a segment is obtained via:

(7) $$\vec{P_{ij}^{\prime }P_{i,j+1}^{\prime }}=\vec{k}\times \left(\vec{P_{ij}P_{i,j+1}}\times \vec{k}\right)$$

The turning angle is then computed as:

(8) $$\phi_{ij}=\arctan\left(\frac{∥\vec{P_{ij}^{\prime }P_{i,j+1}^{\prime }}\times \vec{P_{i,j+1}^{\prime }P_{i,j+2}^{\prime }}∥}{\vec{P_{ij}^{\prime }P_{i,j+1}^{\prime }}.\vec{P_{i,j+1}^{\prime }P_{i,j+2}^{\prime }}}\right).$$

The climbing angle $\psi_{ij}$ measures the inclination of the actual path segment $\vec{P_{ij}^{\prime }P_{i,j+1}^{\prime }}$ relative to its horizontal projection:

(9) $$\psi_{ij}=\arctan\left(\frac{z_{i,j+1}-z_{ij}}{\vec{P_{ij}^{\prime }P_{i,j+1}^{\prime }}}\right)$$

Finally, the smoothing cost $F_{4}$ aggregates penalties for excessive turning and climbing angles:

(10) $$\psi_{ij}=\arctan\left(\frac{z_{i,j+1}-z_{ij}}{\vec{P_{ij}^{\prime }P_{i,j+1}^{\prime }}}\right)$$

where $\varphi_{1}$ and $\varphi_{2}$ are weighting coefficients that penalize sharp turns and steep climbs, respectively.

## 3. Pelican Optimization Algorithm

This section outlines the biological inspiration and mathematical formulation of the Pelican Optimization Algorithm (POA).

### 3.1. Inspiration

The Pelican Optimization Algorithm (POA), proposed by Trojovský et al., is a meta-heuristic method derived from the collective foraging behavior of pelicans. These sizable waterbirds are distinguished by their elongated beaks and expandable throat pouches, which they employ skillfully to capture fish and other small aquatic prey. Adult pelicans typically measure about 150 cm in length, possess wingspans reaching 4 m, and weigh between 1.8 and 2.5 kg. Their anatomical adaptations facilitate efficient hunting in aquatic settings.

In the wild, pelicans frequently hunt cooperatively to improve capture success. They often arrange themselves in linear or semicircular formations to herd schools of fish toward shallow coastal waters. Once the prey is concentrated, the birds use their beaks to scoop up both fish and water into their pouches. By subsequently contracting the pouches and expelling the water, they isolate and consume the captured fish. This coordinated, intelligent hunting strategy exemplifies a natural form of resource optimization [50].

The POA mimics this collective foraging and migratory behavior through a computational framework that incorporates two principal phases, corresponding to the pelicans’ hunting sequence:

(i) Exploration Phase: Pelicans survey the environment and move toward potential prey locations.
(ii) Exploitation Phase: Pelicans hover and wait before executing a targeted capture.

**Figure 4:**
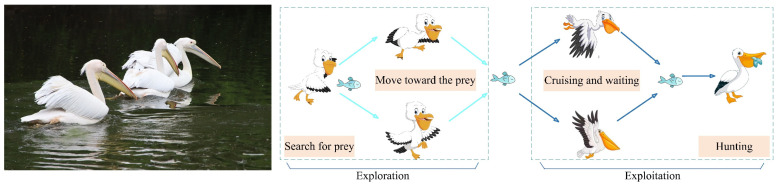
The pelican’s hunting strategy.

These two phases embody the algorithm’s balance between global exploration and local refinement, thereby promoting an effective optimization process. The analogy between pelican hunting behavior and the POA structure is illustrated in Figure 4.

### 3.2. Initialization

In POA, each pelican corresponds to a candidate solution, with its position in the search space determining the initial values of the problem variables. Population initialization is performed randomly within the prescribed bounds according to:

(11) $$X_{i,j}=l_{j}+rand\times \left(u_{j}-l_{j}\right),i=1,2,\ldots ,N,j=1,2,\ldots ,Dim,$$

where $X_{i,j}$ denotes the position of the i-th pelican in the j-th dimension, N is the population size, Dim is the problem dimensionality, randrand is a uniformly distributed random number in [0, 1], and $u_{j}$ and $l_{j}$ represent the upper and lower bounds of the j-th variable, respectively.

The entire population can be represented as a matrix:

(12) $$X={\left[\begin{array}{l} X_{1}\\ \vdots \\ X_{i}\\ \vdots \\ X_{N} \end{array}\right]}_{N\times Dim}={\left[\begin{array}{ccccc} x_{1,1} & \ldots & x_{1,j} & \ldots & x_{1,Dim}\\ \vdots & \ddots & \vdots & ⋰ & \vdots \\ x_{i,1} & \ldots & x_{i,j} & \ldots & x_{i,Dim}\\ \vdots & ⋰ & \vdots & \ddots & \vdots \\ x_{N,1} & \ldots & x_{N,j} & \ldots & x_{N,Dim} \end{array}\right]}_{N\times Dim}$$

where X is the population matrix and $X_{i}$ corresponds to the position vector of the i-th pelican.

Each pelican (i.e., each row of X) encodes a candidate solution. The quality of a solution is evaluated via the objective function, yielding a vector of objective values:

(13) $$F={\left[\begin{array}{c} F_{1}\\ \vdots \\ F_{i}\\ \vdots \\ F_{N} \end{array}\right]}_{N\times 1}={\left[\begin{array}{c} F\left(X_{1}\right)\\ \vdots \\ F\left(X_{i}\right)\\ \vdots \\ F\left(X_{N}\right) \end{array}\right]}_{N\times 1}$$

where F is the objective-function vector and $F_{i}={F(X}_{i})$ represents the fitness of the i-th pelican.

### 3.3. Exploration Stage

During the exploration phase, pelicans navigate toward regions where prey is likely to be found. To emulate this behavior, the prey location is treated as a random point within the search domain. By allowing the prey position to be randomly generated, the algorithm is forced to scan the entire solution space, thereby strengthening its global exploration capacity. This mechanism enables the algorithm to investigate diverse regions and avoid premature convergence.

Mathematically, the movement of a pelican toward a prey item is described by:

(14) $$x_{i,j}^{P1}=\left\{\begin{array}{l} x_{i,j}+rand\times \left(p_{j}-I{\times x}_{i,j}\right),F_{p}<F_{i};\\ x_{i,j}+rand\times \left(x_{i,j}-p_{j}\right),else, \end{array}\right.$$

where $x_{i,j}^{P1}$ denotes the updated position of the i-th pelican in the j-th dimension during the exploration stage; $p_{j}$ represents the prey’s coordinate in the j-th dimension; $F_{p}$ is the objective value at the prey location; and I is a random integer that takes the value 1 or 2, selected independently for each iteration and each individual. The parameter rand is a uniform random number in [0, 1].

If the newly generated position yields a better objective value, the update is retained; otherwise, the pelican remains at its current location. This selective update, termed a “valid update,” prevents the algorithm from drifting toward inferior regions. The acceptance criterion is formalized as:

(15) $$X_{i}=\left\{\begin{array}{ll} X_{i}^{P1}, & F_{i}^{P1}<F_{i};\\ X_{i}, & else, \end{array}\right.$$

where $X_{i}^{P1}$ is the new state vector of the i-th pelican after the exploration update, and $F_{i}^{P1}$ is its corresponding objective value.

### 3.4. Exploitation Stage

Once a prey item has been located, pelicans enter an exploitation phase characterized by hovering, posture adjustment, and a rapid dive toward the water surface. After capturing the prey, they employ wing-beating motions to lift the fish into their throat pouches. Simulating this focused predatory behavior allows the algorithm to intensify its search in promising regions, thereby improving local refinement and solution accuracy.

From a computational perspective, the algorithm conducts a localized search in the vicinity of each pelican’s current position. This behavior is modeled as:

(16) $$x_{i,j}^{P2}=x_{i,j}+R\times \left(1-\frac{t}{T}\right){\times (2\times rand-1)\times x}_{i,j},$$

where $x_{i,j}^{P2}$ is the updated position of the i-th pelican in the j-th dimension during the exploitation stage; R is a constant scaling factor; t is the current iteration index; and T denotes the maximum number of iterations. The term $R\times \left(1-\frac{t}{T}\right)$ functions as an adaptive neighborhood radius that contracts gradually over time, encouraging progressively finer local searches around each individual.

Similar to the exploration phase, a greedy acceptance rule is applied:

(17) $$X_{i}=\left\{\begin{array}{ll} X_{i}^{P2}, & F_{i}^{P2}<F_{i};\\ X_{i}, & else, \end{array}\right.$$

where $X_{i}^{P2}$ is the new state vector after the exploitation update, and $F_{i}^{P2}$ is its associated objective value.

**Figure 5:**
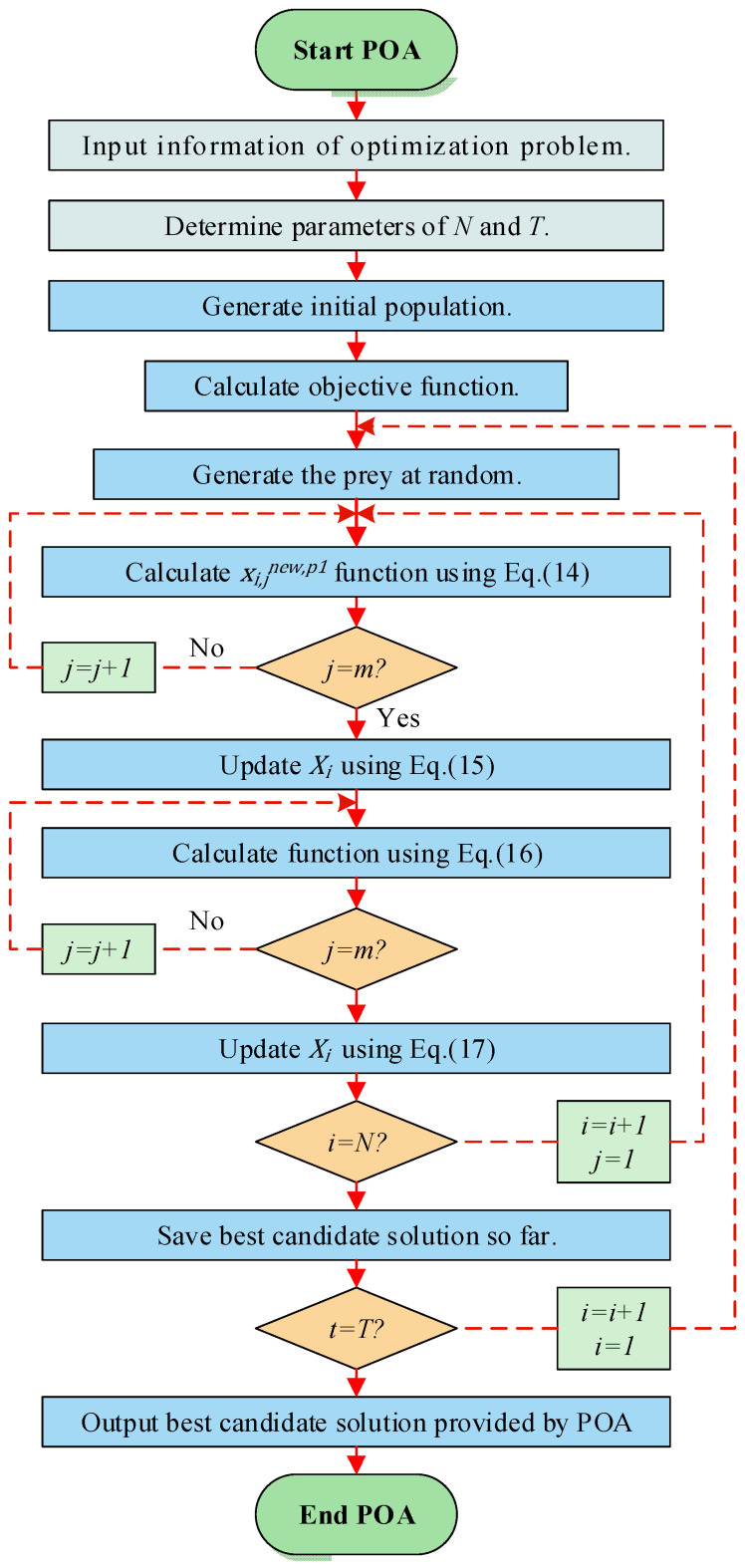
Flowchart of POA.

After all individuals have been updated in both phases, the best candidate solution is re-evaluated based on the new population states and their fitness values. The algorithm then proceeds to the next iteration until the termination criterion is met. The final output is the best solution discovered throughout the run, which serves as an approximate optimum for the given problem. The overall workflow of POA is summarized in the flowchart shown in Figure 5.

## 4. Multi-Strategy Improved Pelican Optimization Algorithm

To overcome the shortcomings of the standard Pelican Optimization Algorithm (POA), such as its limited local and global search performance and insufficient population diversity, this work introduces the Multi-strategy Improved Pelican Optimization Algorithm (MIPOA). MIPOA is constructed by integrating four distinct enhancement mechanisms: a stochastic Lévy-flight strategy, logistic chaotic mapping, an adaptive disturbance factor, and a differential evolution strategy driven by Cauchy mutation.

### 4.1. Logistic Chaotic Mapping

In the original POA, the initialization of candidate solutions often leads to a random and uneven distribution in the search space, which can constrain population diversity and exploration capacity. To address this issue, logistic chaotic mapping is introduced during the population initialization phase [51,52]. Compared with purely random initialization, this deterministic chaotic system generates sequences that exhibit superior ergodicity and pseudo-randomness, thereby promoting a more uniform dispersion of individuals across the feasible region.

The rationale for selecting the logistic map among various chaotic systems stems from its balanced trade-off between ergodicity and randomness, especially when the control parameter λ is set to 3—a value that places the system in a semi-complete chaotic state. While other chaotic maps may excel in specific metrics, the logistic map demonstrates robust overall performance, contributing to improved population diversity without significantly increasing computational overhead. Empirical studies, such as that by Sharma et al., have shown that replacing a standard random-number generator with the logistic chaotic map—coupled with dynamic weight adjustments during the search process—can enhance local search capability and convergence speed, while also reducing average execution time compared to the baseline POA.

**Figure 6:**
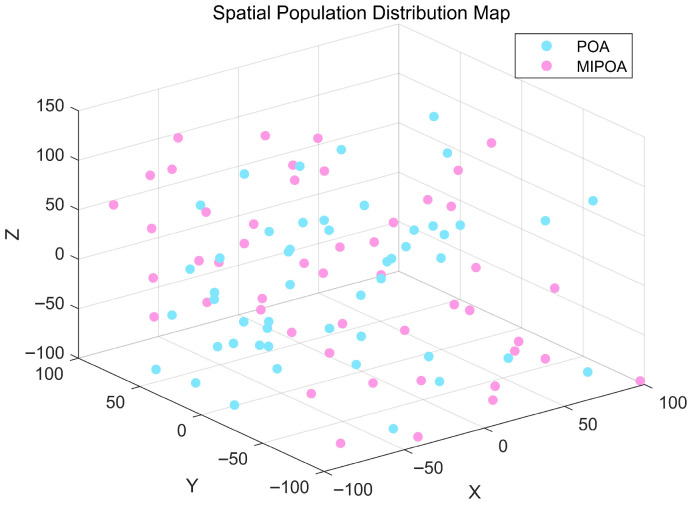
Comparison of population initialization under POA and MIPOA.

The spatial distributions of populations initialized with conventional random sampling (POA) and with logistic chaotic mapping (MIPOA) are contrasted in Figure 6. The visualization clearly indicates that chaotic initialization yields a more homogeneous spread of individuals, which broadens the effective search range and enhances population diversity. Accordingly, the initial position of each pelican in MIPOA is determined by:

(18) $$x_{i,j}=l_{j}+chaos\times \left(u_{j}-l_{j}\right),i=1,2,\ldots ,N,j=1,2,\ldots ,Dim$$

where $x_{i,j}$ denotes the coordinate of the i-th pelican in the j-th dimension; N is the population size; Dim is the problem dimension; $u_{j}$ and $l_{j}$ represent the upper and lower bounds of the j-th variable, respectively; and chaos is a chaotic sequence generated by the logistic map.

The chaotic sequence is computed iteratively as:

(19) $$chaos=y_{n}\left(t+1\right)\times \lambda \times \left(1-y_{n}(t-1)\right)$$

where t is the current iteration index, $y_{n}$ ∈ [0, 1] is the state variable, and λ is the control parameter. While λ is typically set to 4 for fully chaotic behavior, MIPOA adopts λ=3 to achieve a semi-complete chaotic regime that balances exploration thoroughness and algorithmic stability.

### 4.2. Stochastic Lévy Flight Strategy

In metaheuristic optimization, varying step sizes are commonly used in mutation operations to meet different search requirements at different stages of the process. A larger step size is beneficial during early iterations to promote global exploration, enabling the algorithm to rapidly survey the solution space, especially when the initial solutions are far from the optimum. In later stages, a smaller step size facilitates fine-tuning of parameters, allowing the search to converge more precisely toward the optimal value. The complementary use of large and small step sizes thus provides a balanced mechanism for achieving both efficient exploration and stable exploitation.

**Figure 7:**
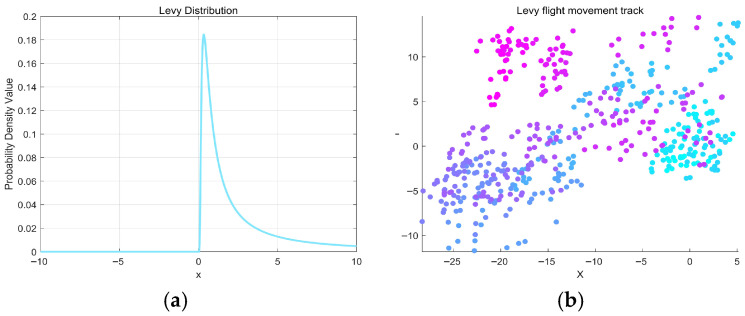
Stochastic Lévy Flight strategy. (**a**) Lévy probability density. (**b**) Stochastic Lévy Flight movement trajectory.

The stochastic Lévy flight adopts a random-walk model whose step lengths follow a heavy-tailed stable distribution, as depicted in Figure 7a. This characteristic enables occasional long-distance jumps while maintaining frequent local steps, which helps the algorithm escape local optima and explore broader regions. MIPOA integrates this stochastic Lévy flight strategy into its exploration phase; typical movement trajectories generated by the strategy are illustrated in Figure 7b.

During the exploration stage of POA, when the condition $F_{P_{i}}<F_{i}$ holds (indicating that the prey location yields a better objective value), the random term randrand in Equation (14) is replaced by a stochastic Lévy-flight step. This modification leverages information from the current best individual to conduct a direct local search around that promising region. Consequently, the position-update formula becomes:

(20) $$x_{i,j}^{\mathrm{new},P1}=\left\{\begin{array}{l} x_{best}+RL\times (p_{j}-I\times x_{i,j}),F_{P_{i}}<F_{i}\\ x_{i,j}+rand\times \left(x_{i,j}-p_{j}\right),{\;\;\;\;\;\;\;\;\;\;\;\;F}_{P_{i}}\geq F_{i} \end{array}\right.$$

where $x_{i,j}^{\mathrm{new},P1}$ is the updated position of the i-th pelican in the j-th dimension after the exploration step; rand is a uniform random number in [0, 1]; $p_{j}$ is the prey coordinate in the j-th dimension; $F_{p}$ denotes the objective value at the prey location; and I is a random integer chosen independently as 1 or 2 for each iteration and each individual. The term $x_{best}$ represents the best solution found before the current iteration, and RL is a weighted Lévy-flight step computed as:

(21) RL=0.5×Levy(Dim)

with Dim being the problem dimensionality and Levy(⋅) the Lévy-flight distribution function, defined by:

(22) $$\mathrm{Levy}\left(D\right)=s\times \frac{u\times \sigma }{|v{|}^{\frac{1}{\eta }}}$$

where s=0.01 and η=1.5 are fixed constants, u and v are independent random numbers drawn uniformly from [0, 1], and σ is given by:

(23) $$\sigma ={\left(\frac{\Gamma \left(1+\eta \right)\times \sin\left(\frac{\pi \eta }{2}\right)}{\Gamma \left(\frac{1+\eta }{2}\right)\times \eta \times 2\left(\frac{\eta -1}{2}\right)}\right)}^{\frac{1}{\eta }}$$

with Γ(⋅) denoting the gamma function.

To prevent the algorithm from moving into inferior regions, the same greedy acceptance criterion as in the original POA is applied. Only if the new position yields a better objective value is the update retained; otherwise, the pelican remains at its previous location. This selective update is expressed as:

(24) $$X_{i}=\left\{\begin{array}{ll} X_{i}^{new,P1}, & F_{i}^{new,P1}<F_{i}\\ X_{i}, & else, \end{array}\right.$$

where $X_{i}^{new,P1}$ is the new state vector of the i-th pelican after the exploration update, and $F_{i}^{new,P1}$ is the corresponding objective value.

### 4.3. Adaptive Disturbance Factor

In the exploitation phase of the original POA, the parameter R in Equation (16) is a fixed constant (set to 0.2). The term $\left(1-\frac{t}{T}\right)$ linearly decays as the iteration index t increases, resulting in a neighborhood radius that decreases uniformly over the entire search process. While such a constant decay rate can guide the search toward local refinement, it may not adequately balance global exploration and fine-tuning across different stages of the optimization.

A more effective approach is to employ an adaptive step-size strategy: a larger step during early iterations accelerates global exploration, especially when initial solutions are far from the optimum; a smaller step in later stages enables precise adjustments, enhancing convergence accuracy and stability. To this end, MIPOA replaces the linear decay factor with an adaptive disturbance factor of the form ${\left(1-\frac{t}{T}\right)}^{\frac{t}{T}}$. This design yields a larger initial radius that decays quickly in the early phase—promoting broad exploration—and then slows its decay rate in later phases—facilitating detailed local search. Compared with linear or exponential decay forms, this nonlinear adaptation better aligns the search behavior with the evolving needs of the optimization process, thereby improving both convergence speed and solution precision.

When the condition rand>0.5 is met during the exploitation stage, the original linear neighborhood radius in Equation (16) is substituted by the adaptive disturbance factor. The corresponding position-update formula becomes:

(25) $$x_{i,j}^{\mathrm{new},P2}=x_{i,j}+\left({\left(1-\frac{t}{T}\right)}^{\frac{t}{T}}\right)\times \left(2\times rand\_1-1\right)\times x_{i,j}$$

where $x_{i,j}^{\mathrm{new},P2}$ denotes the updated position of the i-th pelican in the j-th dimension during exploitation; rand_1 is a random number drawn uniformly from (0.5, 1]; t is the current iteration index; and T is the maximum number of iterations.

Consequently, the neighborhood radius in MIPOA starts at 1 (promoting rapid global exploration) and decreases gradually as the run progresses, with the decay rate slowing down over time. This behavior enables a smooth transition from coarse global scanning to fine-grained local refinement.

### 4.4. Cauchy Mutation-Based Differential Evolution Strategy

**Figure 8:**
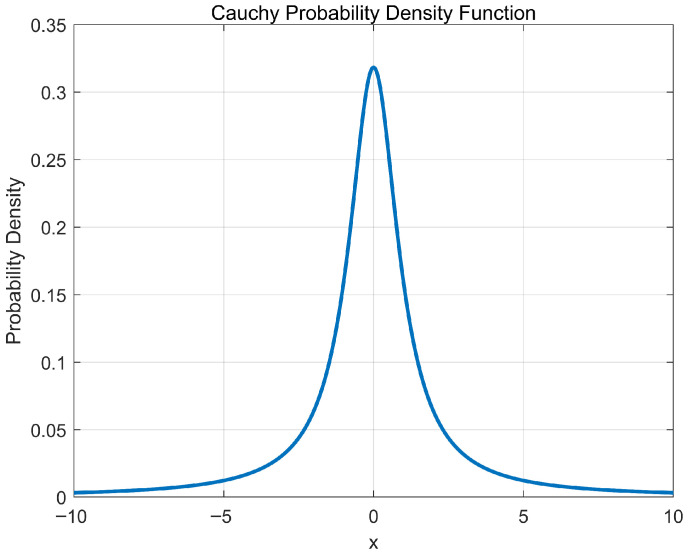
Probability density function of the Cauchy distribution.

The standard POA still exhibits a tendency to stagnate in local optima during later iterations. To mitigate this issue, MIPOA further incorporates a Cauchy-mutation-based differential evolution strategy in its exploitation phase. The Cauchy distribution, a continuous probability distribution with a sharp peak at the origin and long, flat tails (Figure 8), is defined by the probability density function:

(26) $$f\left(x\right)=\frac{1}{\pi \left(x^{2}+1\right)},x\;\in (-\infty ,+\infty )$$

The heavy-tailed nature of the Cauchy distribution endows its mutation operator with strong perturbation capability. By generating occasional large jumps while maintaining frequent small steps, Cauchy mutation helps the algorithm escape local basins and explore broader regions, thereby reducing the risk of premature convergence.

In the original POA exploitation stage, Equation (16) only considers a single random individual and searches within a fixed neighborhood radius. In contrast, the differential-evolution-inspired approach adopted here selects three distinct random individuals from the current population and performs a perturbation based on the difference vector between two of them. The Cauchy mutation operator is computed as:

(27) $$Cauchy=\tan\left(0.5\times \pi \times \left(rand\_2-0.5\right)\right)$$

where rand_2 is a uniform random number in [0,0.5]. The position update is then given by:

(28) $$x_{i,j}^{\mathrm{new},P2}=x_{i,j}+Cauchy\times \left(x_{random\_1}-x_{random\_2}\right)$$

in which $x_{random\_1}$ and $x_{random\_2}$ are two distinct random candidate solutions drawn from the current population during the exploitation phase, and $x_{i,j}^{\mathrm{new},P2}$ is the resulting perturbed position.

By integrating this Cauchy-mutation-based differential evolution strategy, MIPOA injects additional diversity into the population. The use of difference vectors combined with Cauchy-distributed perturbations makes the mutation patterns more varied across generations, which helps the algorithm avoid local traps and strengthens its global search ability.

### 4.5. Enhanced Exploitation Strategy

To balance the application of the two exploitation mechanisms introduced in Section 4.3 and Section 4.4, a switching probability $R_{1}=0.5$ is defined. During the exploitation phase of MIPOA, a uniform random number rand∈[0,1] is generated for each individual. If $rand>R_{1}$ (i.e., rand_1∈(0.5,1]), the position update follows the adaptive-disturbance strategy given by Equation (25). Otherwise, when $rand\leq R_{1}$ (i.e., rand_2∈[0,0.5]), the update is performed using the Cauchy-mutation-based differential evolution strategy defined in Equation (28). This probabilistic switching ensures that both local refinement and diversity-preserving perturbation are utilized in a complementary manner throughout the exploitation stage.

Mathematically, the combined exploitation-phase position update is expressed as:

(29) $$x_{i,j}^{\mathrm{new},P2}=\left\{\begin{array}{l} x_{i,j}+Cauchy\times \left(x_{random\_1}-x_{random\_2}\right),rand>R_{1}\\ x_{i,j}+\left({\left(1-\frac{t}{T}\right)}^{\frac{t}{T}}\right)\times \left(2\times rand_{1}-1\right)\times x_{i,j},else \end{array}\right.$$

To maintain solution quality, a greedy acceptance criterion is applied after each exploitation update. The new position is retained only if it yields an improved objective value; otherwise, the individual remains at its previous location. This selection rule is formalized as:

(30) Xi=Xinew,P2,F_i^(new,P2)<Fi;Xi,else,

where $X_{i}^{new,P2}$ denotes the new state vector of the i-th pelican after the exploitation update, and $F_{i}^{new,P2}$ is the corresponding objective-function value.

The complete workflow of the proposed MIPOA is summarized in Algorithm 1.
**Algorithm 1** Pseudo Code of MIPOAStart MIPOA. 1: Input information of optimization problem. 2: Determine the MIPOA population _size
(N) and the number of
$\left(T\right)$. 3: Initialize problem setting (
Dim,
lb,
ub). 4: Initialize of the position of pelicans using Equation (18) and calculate the objective function. 5: **For** 
t=1:T
6:     Generate the position of the prey at random. 7:     **For** 
i=1:N
8:     Phase 1: Search for prey and move towards it (exploration phase). 9:         **For** 
j=1:m 10:          Calculate new status of the
j*-th* dimension using Equation (20). 11:        End. 10:        update the position of
ith pelican using Equation (24). 12:    Phase 2: Cruising and waiting, and hunting prey (exploitation phase). 13:        **For** 
j=1:m
14:          Calculate Cauchy using Equation (27). 15:          if
rand>0.5
16:           Calculate new status of the
jth dimension using Equation (25). 17:          else 18:           Calculate new status of the
jth dimension using Equation (28). 19:        End 20:        update the position of
ith pelican using Equation (29). 21:   End. 22:   Update best candidate solution. 23: End. 24: Output best candidate solution obtained by MIPOA. End MIPOA.

## 5. Experimental Results and Analysis

To quantitatively assess the performance of the proposed Multi-strategy Improved Pelican Optimization Algorithm (MIPOA) and examine its problem-solving effectiveness, this section reports three sets of experiments. A comparative statistical analysis between MIPOA and a suite of well-established metaheuristic algorithms is carried out. First, the algorithm’s fundamental characteristics—including its exploration-exploitation balance and population diversity—are evaluated. Second, MIPOA is benchmarked on the widely adopted CEC-2017 and CEC-2022 test suite [53,54], where it is compared against twelve state-of-the-art optimizers. The results are systematically analyzed to provide a comprehensive performance assessment. Finally, a non-parametric Friedman ranking test is conducted, with the mean objective value and its standard deviation serving as the primary evaluation metrics.

The following algorithms are included in the comparison: AVOA [36], DBO [37], GSA [30], GWO [31], AO [38], MFO [32], WOA [33], HHO [39], SO [34], CPSOGSA [55], CSA [56] and POA [57]. The parameter configurations of all competing algorithms are summarized in Table 1. Each algorithm was executed independently 30 times under identical conditions to ensure statistical reliability. In the subsequent result tables, the best outcome for each test function and dimension is emphasized in bold.

The experimental configuration for evaluating MIPOA is as follows. All algorithms are independently executed 30 times to ensure statistical robustness. For the CEC-2017 benchmark tests, the maximum number of iterations is fixed at 500, with problem dimensions set to 30, 50, and 100, respectively. In all trials, the population size (i.e., the number of search agents) is maintained at 30 for every algorithm. MIPOA is implemented in MATLAB, and all simulations are performed on a desktop computer running Windows 10, equipped with an Intel Core i5 processor operating at 2.5 GHz and 16 GB of RAM.

### 5.1. Convergence Behavior Analysis

**Figure 9:**
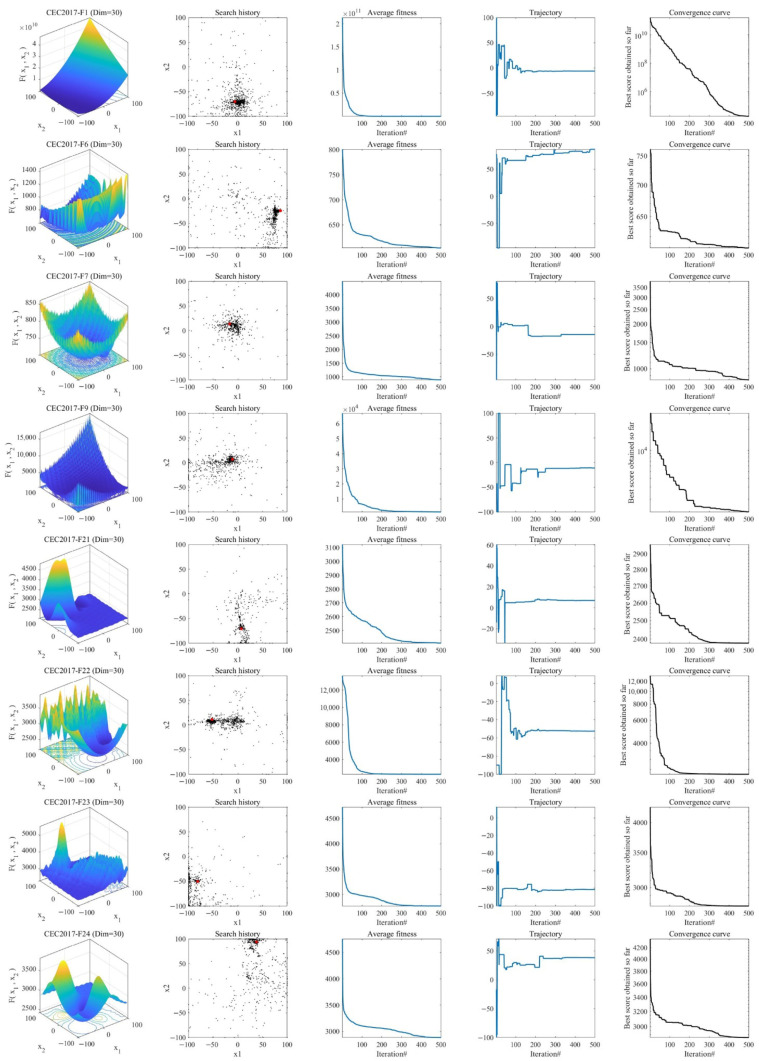
Convergence behavior of MIPOA.

To investigate the convergence properties of MIPOA, this subsection provides a visual and quantitative examination of its search dynamics. As depicted in Figure 9, the first column displays two-dimensional contour maps of selected test functions, highlighting their multimodal and complex landscapes. The second column illustrates the spatial distribution of the search agents during an intermediate iteration. It can be observed that most individuals concentrate near the global optimum, while a subset remains widely dispersed across the search space—a pattern that reflects the algorithm’s effective balance between exploitation and exploration, and its ability to avoid premature convergence.

The third column traces the evolution of the population’s average fitness over iterations. In early stages, the average fitness remains relatively high, indicating that the agents are broadly exploring the search domain. As iterations proceed, the average fitness declines sharply, demonstrating the algorithm’s capacity to rapidly direct the population toward promising regions.

The fourth column portrays the search trajectories of several representative individuals. These trajectories transition from irregular, wide-ranging movements in the initial phase to stable, localized oscillations in later phases, illustrating a smooth shift from global exploration to focused local refinement.

Finally, the fifth column presents the convergence curves of MIPOA on both unimodal and multimodal functions. For unimodal functions, the curve exhibits a steep, monotonic descent, reflecting fast initial convergence that gradually slows as the solution approaches the optimum. For multimodal functions, the curve follows a stepwise descending pattern, indicating periodic escapes from local basins and sustained progress toward the global optimum. Collectively, these observations confirm that MIPOA maintains strong global exploration while achieving precise local convergence, thereby consistently attaining high-quality solutions.

### 5.2. Experimental Evaluation on CEC-2017 Benchmark Functions

The CEC-2017 benchmark suite is composed of four categories: unimodal functions, multimodal functions, hybrid functions, and composite functions. Unimodal functions possess a single global optimum without local traps, making them suitable for evaluating an algorithm’s exploitation ability. Multimodal functions contain numerous local optima and are primarily used to test the algorithm’s capacity to locate the global optimum and escape from deceptive basins. Hybrid and composite functions further increase complexity by combining different function characteristics, thus assessing the algorithm’s robustness in handling intricate continuous optimization problems.

**Figure 10:**
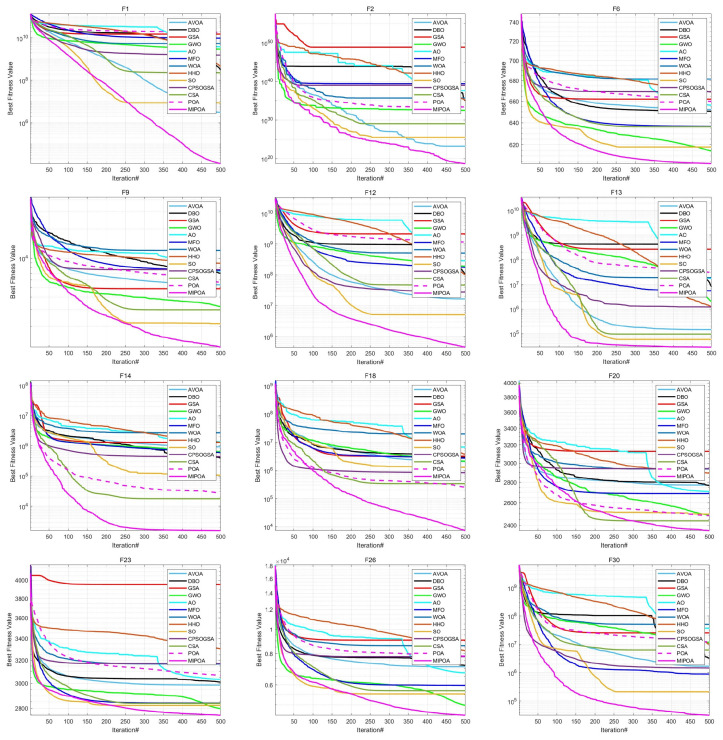
Convergence curves on CEC-2017 test functions (Dim = 30).

**Figure 11:**
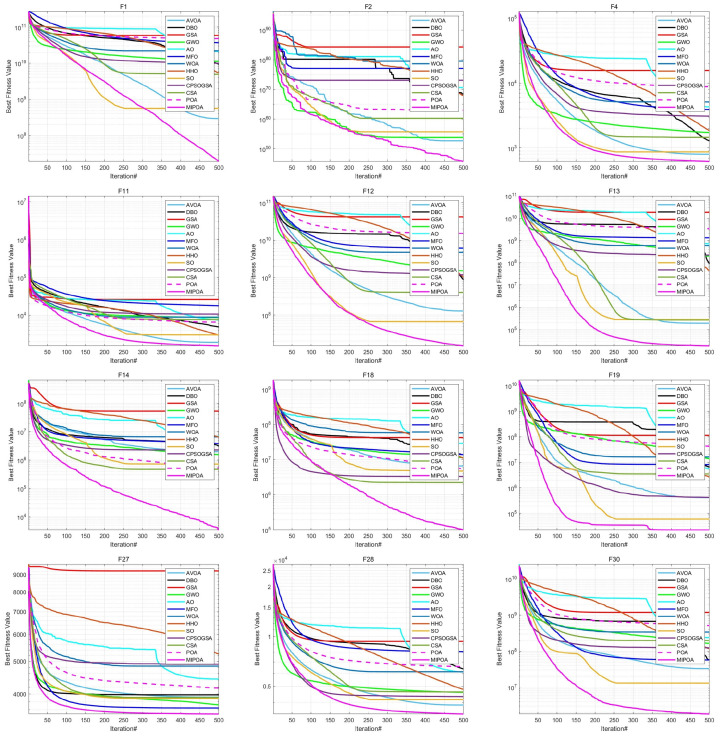
Convergence curves on CEC-2017 test functions (Dim = 50).

**Figure 12:**
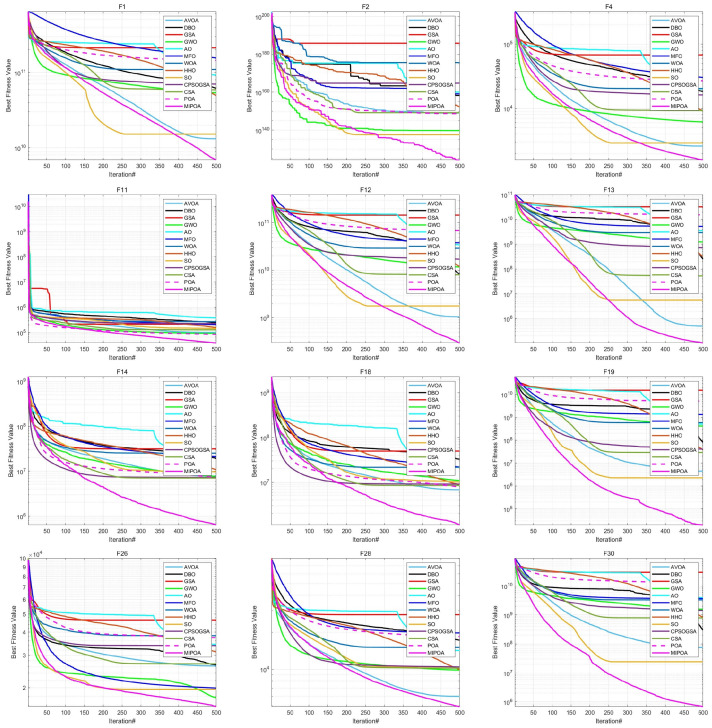
Convergence curves on CEC-2017 test functions (Dim = 100).

The comparative results of MIPOA against the twelve competitor algorithms on the CEC-2017 functions at dimensions 30, 50, and 100 are summarized in Table 2, Table 3 and Table 4 (tabular data are provided in the manuscript), with the best values displayed in bold. Representative convergence curves for selected functions are shown in Figure 10, Figure 11 and Figure 12. The results demonstrate that MIPOA consistently attains the highest number of top-ranked performances and the best average ranking across all three dimensions.

Specifically, in the 30-dimensional tests, MIPOA achieved the best average value on 26 out of 30 functions, secured the third-best average on three functions, and ranked twelfth on one function, with no instance of the lowest ranking. As dimensionality increases to 50 and 100, MIPOA continues to exhibit strong optimization performance, obtaining the optimal average value on 40 functions across the two dimensional settings. Its detailed ranking distribution is as follows: second place on one function, third on seven, fourth on one, fifth on four, sixth on one, seventh on one, tenth on one, twelfth on three, and thirteenth on one.

**Figure 13:**
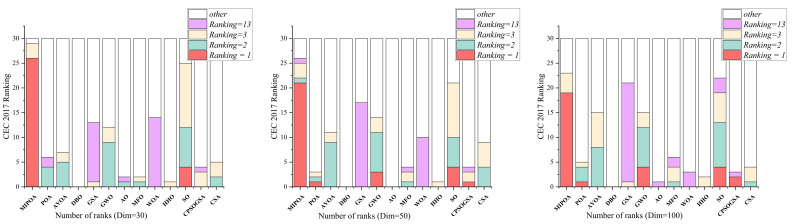
Ranking comparison of algorithms on CEC-2017 test functions.

Figure 13 illustrates the ranking distribution of MIPOA relative to the twelve advanced algorithms across the three dimensions. To highlight its superiority, the ranking outcomes are grouped into five categories: first place, second place, third place, worst rank, and other positions. The figure clearly shows that MIPOA dominates in both the count of first-place finishes and the overall average rank, with minimal variation across dimensions—reflecting not only excellent optimization capability but also high stability.

In terms of the Friedman rank test, recently proposed algorithms such as AVOA and DBO achieve Friedman scores of 5.50 and 7.43, respectively, which are superior to those of classical algorithms like GSA (10.43) and WOA (11.93). This suggests that newer metaheuristics generally offer better performance on this benchmark set. Notably, MIPOA attains a Friedman score of 1.57, outperforming all competitors. In the 50- and 100-dimensional cases, MIPOA again achieves the lowest (best) Friedman values, confirming that the integrated strategies—chaotic initialization, Lévy-flight exploration, adaptive disturbance, and Cauchy-based differential evolution—collectively enhance the algorithm’s exploration capacity and overall efficacy.

Furthermore, the convergence curves (Figure 10, Figure 11 and Figure 12) reveal that several algorithms, including SO, WOA, AO, HHO, and the original POA, tend to stagnate in local optima during later iterations and exhibit limited ability to escape across different dimensions. In contrast, MIPOA maintains vigorous search activity even in the final stages. Although MIPOA temporarily converged to local basins on functions F2, F18, F19, and F23, it successfully broke away near the end of the runs, resuming deep global exploration and ultimately attaining high-precision solutions. These observations indicate that the adaptive disturbance factor and the stochastic Lévy-flight strategy not only help MIPOA evade local traps but also accelerate convergence and improve solution accuracy.

### 5.3. Experimental Evaluation on CEC-2022 Benchmark Functions

To further assess the scalability and robustness of the proposed algorithm, MIPOA is also validated on the more recent CEC-2022 benchmark suite. As with CEC-2017, this test set comprises unimodal, multimodal, hybrid, and composite functions [58], offering a challenging platform for evaluating advanced optimization techniques. In this experiment, each algorithm is executed 12 independent runs with a maximum of 500 iterations, and the problem dimension is fixed at 20.

**Figure 14:**
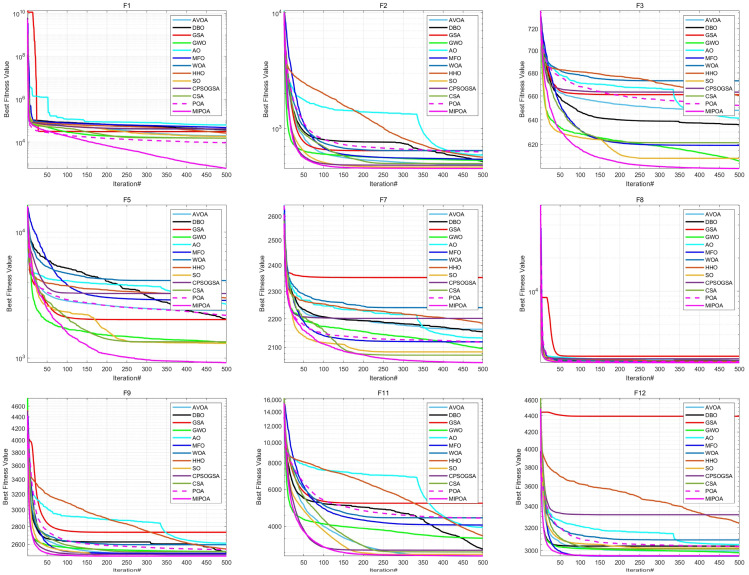
Convergence curves on CEC-2022 test functions (Dim = 20).

The comparative results of MIPOA against the twelve competitor algorithms on the CEC-2022 functions are summarized in Table 5 (provided in the manuscript). Convergence trajectories for representative functions are plotted in Figure 14. The experimental outcomes reveal that MIPOA achieves the best average ranking on 7 out of 12 functions, the second-best average on 3 functions, the third-best on 1 function, and the fourth-best on 1 function.

**Figure 15:**
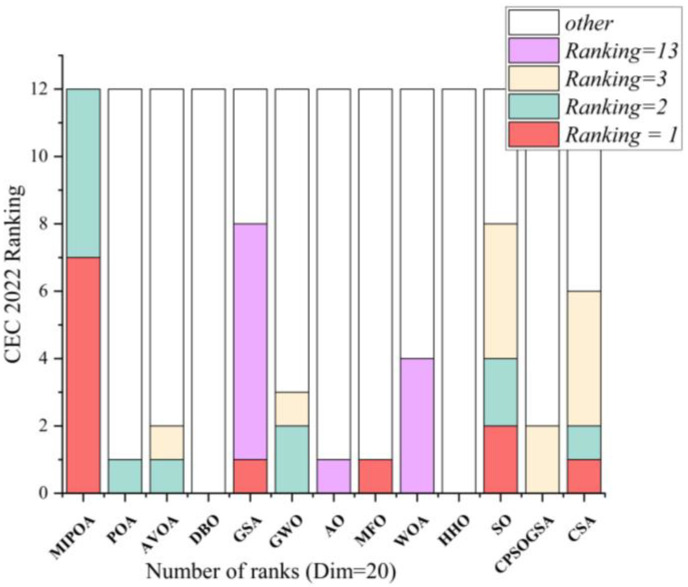
Ranking comparison of algorithms on CEC-2022 test functions.

To visually contrast the ranking profiles of all algorithms, a stacked bar chart is presented in Figure 15. The chart clearly shows that MIPOA never attains the worst rank on any function, underscoring its consistent effectiveness and strong scalability. In terms of overall composite ranking, MIPOA occupies the top position and outperforms the other methods by a considerable margin.

An analysis of the convergence curves in Figure 14 indicates that MIPOA converges more rapidly and reaches higher-precision solutions than the competing algorithms. This accelerated and accurate convergence can be attributed to the synergistic action of the adaptive disturbance factor and the Cauchy-mutation-based differential evolution strategy, which together enhance both the speed and precision of the search process.

### 5.4. Ablation Experiment Analysis

To isolate the contribution of each improvement component, an ablation study was conducted. Four variants of the baseline Pelican Optimization Algorithm (POA) were constructed by individually incorporating the following strategies: Logistic Chaotic Mapping (LCM-POA), Stochastic Lévy Flight (SLF-POA), Adaptive Disturbance Factor (ADF-POA), and Cauchy-Mutation-based Differential Evolution (CMDE-POA).

**Figure 16:**
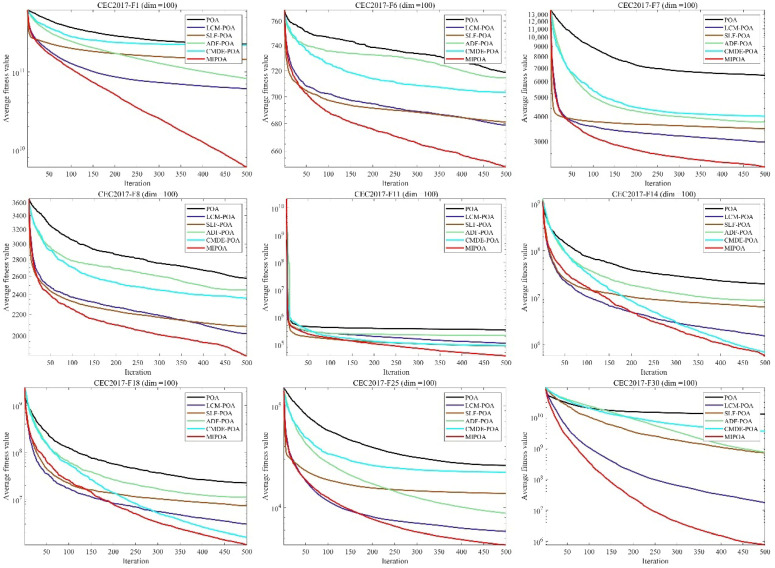
Performance comparison of different strategy variants.

As illustrated in Figure 16, each variant improves the convergence speed and solution accuracy of the original POA to varying degrees. The integrated MIPOA, which combines all four strategies, delivers the most pronounced enhancement. On unimodal and multimodal functions, the performances of ADF-POA and CMDE-POA are relatively similar, while LCM-POA and SLF-POA exhibit more substantial gains. For more complex hybrid and composite functions, the effectiveness of LCM-POA and ADF-POA diminishes, whereas SLF-POA and CMDE-POA retain stronger performance, underscoring their role in escaping deceptive local basins. Overall, MIPOA consistently outperforms all individual variants across the majority of test functions, confirming the synergistic benefits of the multi-strategy integration. Consequently, MIPOA effectively mitigates premature convergence and local-optima stagnation, leading to accelerated convergence and improved solution precision.

### 5.5. Parameter Sensitivity Analysis

In Equation (29), the balance factor $R_{1}$ is set to 0.5 to ensure equal probability between the two exploitation strategies (adaptive disturbance factor and Cauchy-mutation-based differential evolution). To verify the appropriateness of this choice, a sensitivity analysis was performed by varying $R_{1}$ across the set {0, 0.1, 0.2, 0.3, 0.4, 0.5, 0.6, 0.7, 0.8, 0.9, 1}. For each value, MIPOA was executed independently 30 times on all 30 CEC-2017 functions, and the average ranking across functions was used as the performance metric.

**Figure 17:**
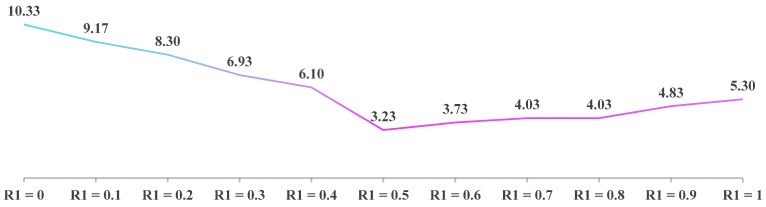
Sensitivity of MIPOA performance to the balance factor $R_{1}$.

The resulting average rankings are plotted in Figure 17. The curve indicates that $R_{1}=0.5$ yields the best overall performance, achieving the lowest average rank of 3.23. The second-best setting, $R_{1}=0.6$, produces a higher average rank of 3.73. Hence, $R_{1}=0.5$ is adopted as the optimal value to balance exploitation diversity and algorithmic robustness.

### 5.6. Statistical Significance Test

To rigorously compare the performance of MIPOA against the twelve competitor algorithms, a non-parametric Wilcoxon rank-sum test was conducted at a 5% significance level. A p-value lower than 0.05 rejects the null hypothesis, indicating a statistically significant difference between the two algorithms; conversely, a p-value above 0.05 suggests that the algorithms do not differ significantly in optimization capability [59,60,61]. The test outcomes are listed in Table 6, where entries with p>0.05 are highlighted in bold. Such cases are extremely rare, providing strong evidence that MIPOA significantly outperforms the other algorithms on the CEC-2017 benchmark suite.

Furthermore, by combining the observed performance metrics with the Wilcoxon test results, it can be concluded that MIPOA not only exhibits superior optimization efficacy but also maintains broad applicability across diverse problem types. This versatility stems from the synergistic effects of the four novel strategies—chaotic initialization, Lévy-flight exploration, adaptive disturbance, and Cauchy-based differential evolution—which collectively enhance the algorithm’s ability to tackle complex optimization challenges.

### 5.7. Computational Complexity Analysis

This section outlines the computational complexity of the proposed MIPOA. As is typical for swarm-intelligence methods, the complexity is influenced by implementation details, problem characteristics, and parameter choices. For MIPOA, the primary computational overhead arises from four operations: population initialization, objective-function evaluation, prey-position generation, and iterative solution updates.

Using big- O notation, let N denote the population size, T the maximum iteration count, and m the problem dimension. The initialization step requires O(N) operations. In each iteration, every individual performs one fitness evaluation during the exploration phase and another during the exploitation phase, leading to a total of O(2×T×N) function evaluations. Prey generation (which involves random sampling and evaluation) is performed once per iteration, contributing $O\left(T\right)+O(T\times N)$ operations. Updating the positions of all N individuals across both phases involves processing *m* dimensions per individual, resulting in an update complexity of O(2×T×N×m). Aggregating these components, the overall computational complexity of MIPOA can be expressed as $O(N+T\times \left(1+m\right)\times \left(1+2N\right))$. Since MIPOA does not introduce additional iterative loops beyond those of the baseline POA, its asymptotic complexity remains on the same order as that of the original algorithm.

**Figure 18:**
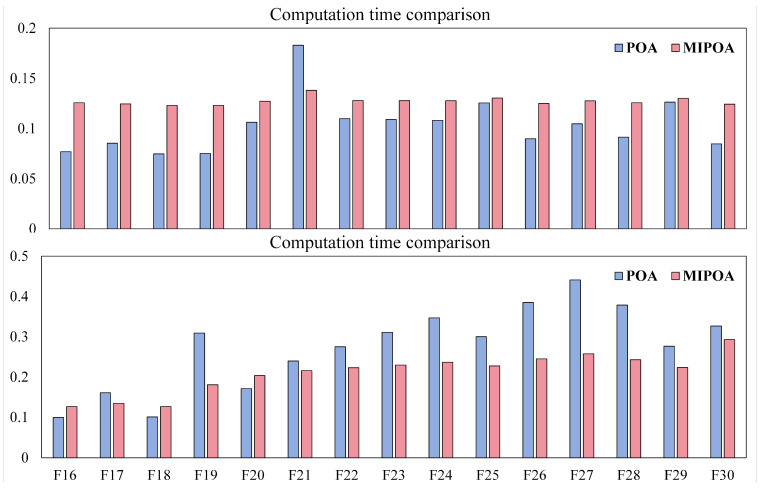
Runtime comparison between MIPOA and POA on CEC-2017 functions.

To empirically assess the actual runtime overhead, MIPOA and POA were independently executed on the CEC-2017 benchmark suite, and the average execution times per function are plotted in Figure 18. The figure reveals that on a subset of functions (e.g., F6, F17, F19, and F21) MIPOA incurs slightly lower computational cost than POA. However, on the majority of functions, MIPOA exhibits a modest increase in runtime. This increase is attributed to the extra operations introduced by the four enhancement strategies. Despite the slight rise in computational cost, MIPOA delivers substantially superior optimization accuracy and convergence speed, achieving a favorable trade-off between computational efficiency and performance improvement.

## 6. MIPOA for 3D UAV Path Planning

### 6.1. Scenario Settings

**Figure 19:**
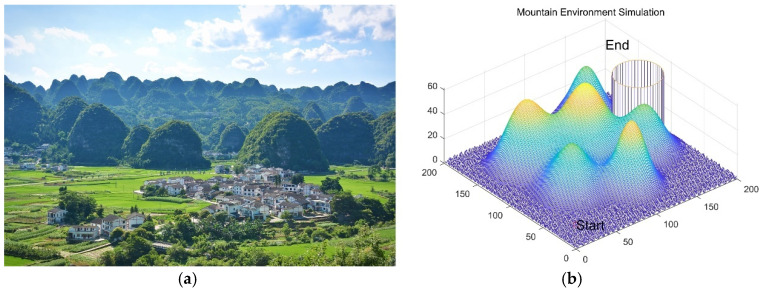
Simulation environment model. (**a**) Wanfenglin Mountain area. (**b**) Simulation environment model.

The experimental scenario is based on the karst mountain terrain of the Wanfenglin area in Guizhou, China—a region characterized by complex topography with numerous peaks, rugged terrain, and scattered residential zones (Figure 19a). Karst landscapes pose significant challenges for UAV path planning due to irregular elevations, restricted airspace, and variable meteorological conditions [45]. To ensure operational safety, flight trajectories must circumvent hazardous features such as steep slopes, narrow valleys, and designated no-fly zones. Therefore, the planning model incorporates terrain elevation, static obstacles, and prohibited areas as critical constraints.

The simulation environment is mathematically modeled by the elevation function:

(31) $$z=sin(y+1)+sin(x)+cos(x^{2}+y^{2})+2\times cos(y)+sin(x^{2}+y^{2})$$

which generates a realistic, undulating surface representing the karst landscape (Figure 19b).

### 6.2. Competing Algorithms and Parameter Settings

To assess the spatial-planning capability of MIPOA, the same twelve meta-heuristic algorithms used in the preceding benchmark tests are employed for comparison. The experimental setup for 3D UAV path planning is configured as follows: UAV cruise speed is fixed at 20 m/s, maximum flight altitude $H_{max}=50$ m, and maximum turning angle φ=60°. The operational airspace is defined as a 200 m × 200 m × 20 m volume. The UAV starts at coordinates (0, 0, 20) and aims to reach the target (200, 200, 30). For all thirteen algorithms, the population size is set to 50 and the maximum iteration count to 500. To mitigate random fluctuations, each algorithm is executed independently 30 times under identical conditions.

### 6.3. Simulation and Analysis

**Figure 20:**
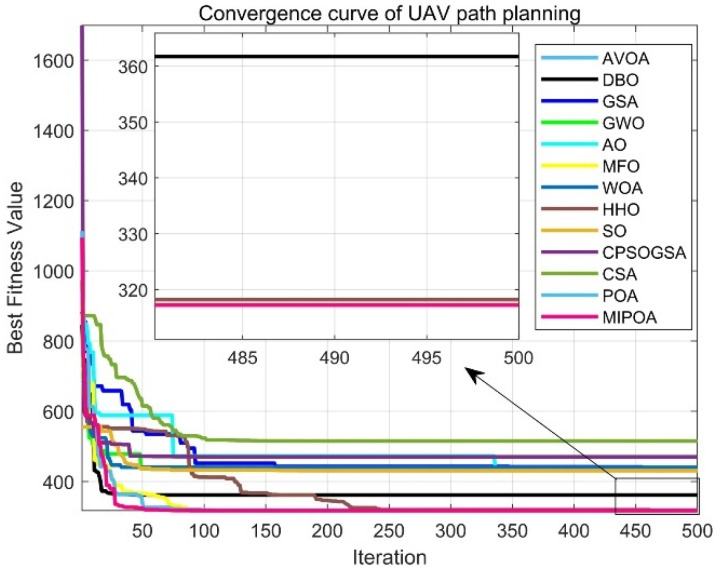
Convergence curves of different algorithms for UAV path planning.

**Figure 21:**
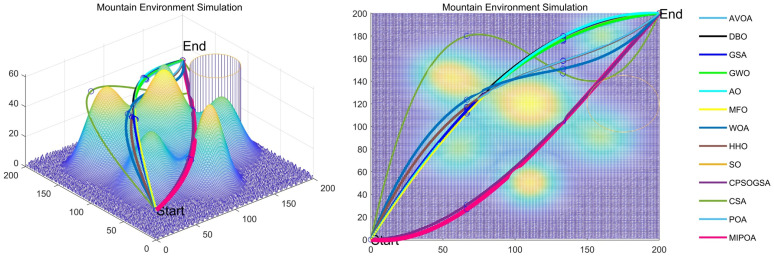
Three-dimensional and two-dimensional views of the planned optimal paths.

The path-planning results obtained by MIPOA and the twelve competitor algorithms are summarized in Table 7, where “Best” denotes the shortest path found, “Ave” the mean path length over 30 runs, and “Std” the corresponding standard deviation. The convergence histories during the search process are plotted in Figure 20, while the resulting 3D and 2D trajectory visualizations are provided in Figure 21.

As indicated in Table 7, MIPOA achieves both the smallest best path length and the lowest average path length, demonstrating superior solution accuracy. Notably, MIPOA’s average path length is even shorter than the best paths produced by most other algorithms. Although MIPOA ranks third in terms of standard deviation, its performance exhibits greater stability compared to the original POA and several other methods.

From the convergence curves in Figure 20, MIPOA exhibits a steeper and more rapid decline in path cost, confirming its faster convergence rate and higher optimization precision relative to POA and the other competitors. The trajectory plots in Figure 21 show that all algorithms successfully avoid designated threat zones, verifying the feasibility of their solutions. However, MIPOA generates the shortest and smoothest trajectory, whereas CSA yields the longest path, necessitating pronounced altitude adjustments to circumvent mountainous obstacles.

In summary, the experimental outcomes validate the effectiveness of MIPOA for 3D UAV path planning in complex terrains. Its ability to produce shorter, smoother, and computationally stable trajectories underscores its practical potential for a wide range of UAV applications.

## 7. Summary and Prospect

This work addresses the principal shortcomings of the Pelican Optimization Algorithm (POA), especially its susceptibility to local optima and limited population diversity, by introducing a multi-strategy improved variant termed MIPOA. The proposed enhancements systematically target four key aspects: population initialization, individual diversity, global exploration, and local exploitation.

During initialization, logistic chaotic mapping is adopted to produce a more uniformly distributed population, thereby broadening the initial search coverage. In the exploration phase, a stochastic Lévy-flight mechanism is embedded to strengthen global scanning while preserving diversity. For the exploitation phase, an adaptive disturbance factor is designed to accelerate convergence and refine solution accuracy; concurrently, a differential evolution strategy driven by Cauchy mutation is incorporated to inject additional population diversity. The synergistic integration of these strategies collectively elevates the convergence capability of MIPOA.

The performance of MIPOA was rigorously assessed through extensive experiments. On the CEC-2017 and CEC-2022 benchmark suites, MIPOA exhibited faster convergence and higher solution precision compared with twelve state-of-the-art meta-heuristic algorithms. The Friedman ranking tests further substantiated its statistical superiority. In a practical 3D UAV path-planning scenario, MIPOA consistently delivered shorter, smoother, and more stable trajectories than the competing methods, demonstrating its effectiveness in real-world applications.

Although MIPOA has shown clear advantages in global optimization and UAV path planning, further refinements are warranted in light of the No-Free-Lunch theorem. Future research will extend MIPOA to broader domains, including UAV-swarm cooperative control, neural-network hyperparameter tuning, and digital-twin modeling of human–machine systems [54]. Concurrently, algorithmic improvements will focus on more adaptive initialization schemes and enhanced evolutionary operators to boost robustness and adaptability.

Looking ahead, MIPOA holds considerable potential beyond UAV trajectory planning. Its strong robustness and high convergence accuracy make it a promising candidate for complex system optimization in fields such as intelligent manufacturing, smart transportation, and disaster monitoring. Two immediate directions for extension are (1) dynamic-environment path planning, where real-time environment updates, predictive models, or receding-horizon strategies would enable adaptation to moving obstacles and changing terrains; and (2) multi-UAV cooperative planning, in which coordination mechanisms, task-allocation policies, and communication-constrained models would allow MIPOA to tackle formation flying, collaborative reconnaissance, and logistics drone fleet operations.

## Figures and Tables

**Table 1:** Parameter settings of the compared algorithms.

Algorithms	Name of the Parameter	Value of the Parameter
AVOA	L1, L2, w, p1, p2, p3	0.8, 0.2, 2.5, 0.6, 0.4, 0.6
DBO	P_percent	0.2
GSA	ElitistCheck, min_flag, Rpower, Rnorm	1, 1, 1, 2
GWO	a	[0, 2]
AO	alpha, delta	0.1, 0.1
MFO	b, t	1, [−1, 1]
WOA	a, a2, b	[0, 2], [−1, −2], 1
HHO	E0, E1	[−1, 1], [0, 2]
SO	Q, T, c1, c2, c3	0.25, 0.6, 0.5, 0.05, 2
CPSOGSA	φ1, φ2	2.05, 2.05
CSA	γ, α, β	1, 3.5, 3
POA	I, r, R	1 or 2, [0, 1], 0.2
MIPOA	$y_{n},\lambda ,\lambda$$,\;I,\;r,\;R,\;R_{1}$, rand_1, br-to-break rand_2	[0, 1], [1, 4], 4, 1 or 2, [0, 1], 0.2, 0.5, (0.5, 1], [0.5, 1]

**Table 2:** Experimental results on CEC-2017 test functions (Dim = 30).

ID		AVOA	DBO	GSA	GWO	AO	MFO	WOA	HHO	SO	CPSOGSA	CSA	POA	MIPOA
F1	Ave	3.1505 × 10^6^	3.1434 × 10^8^	1.5099 × 10^10^	2.9710 × 10^9^	3.8372 × 10^9^	9.8246 × 10^9^	5.4450 × 10^9^	4.6115 × 10^8^	8.7570 × 10^6^	1.5648 × 10^9^	2.2961 × 10^8^	1.8596 × 10^10^	**1.1802 × 10^4^**
	Std	1.0203 × 10^7^	1.6773 × 10^8^	3.9384 × 10^9^	1.8918 × 10^9^	1.3261 × 10^9^	7.2579 × 10^9^	2.1448 × 10^9^	3.4674 × 10^8^	8.1165 × 10^6^	3.2804 × 10^9^	1.5624 × 10^8^	6.3040 × 10^9^	**7.7758 × 10^3^**
F2	Ave	1.7789 × 10^23^	1.1244 × 10^35^	7.5184 × 10^48^	3.4715 × 10^32^	4.2623 × 10^37^	2.6699 × 10^39^	5.1952 × 10^35^	1.7237 × 10^35^	3.3570 × 10^25^	1.0746 × 10^39^	1.1981 × 10^29^	2.6096 × 10^33^	**5.3306 × 10^18^**
	Std	4.8839 × 10^23^	6.0917 × 10^35^	2.7929 × 10^49^	1.2657 × 10^33^	1.6188 × 10^38^	1.4593 × 10^40^	2.6122 × 10^36^	8.5022 × 10^35^	1.2387 × 10^26^	4.8941 × 10^39^	4.0585 × 10^29^	6.8741 × 10^33^	**1.1197 × 10^19^**
F3	Ave	5.5768 × 10^4^	9.3627 × 10^4^	9.6585 × 10^4^	6.1433 × 10^4^	6.8587 × 10^4^	1.5969 × 10^5^	2.5883 × 10^5^	5.3973 × 10^4^	6.9610 × 10^4^	1.7081 × 10^5^	7.8758 × 10^4^	4.1442 × 10^4^	**1.4931 × 10^4^**
	Std	8.3457 × 10^3^	2.8661 × 10^4^	1.1772 × 10^4^	1.0935 × 10^4^	9.7769 × 10^3^	4.7862 × 10^4^	4.9127 × 10^4^	7.3255 × 10^3^	9.8252 × 10^3^	4.8702 × 10^4^	2.2477 × 10^4^	7.2396 × 10^3^	**5.4260 × 10^3^**
F4	Ave	5.3321 × 10^2^	6.6225 × 10^2^	3.4501 × 10^3^	6.4978 × 10^2^	1.1058 × 10^3^	1.1521 × 10^3^	1.2117 × 10^3^	7.4470 × 10^2^	5.7505 × 10^2^	7.7391 × 10^2^	5.8196 × 10^2^	2.0773 × 10^3^	**4.9458 × 10^2^**
	Std	2.9313 × 10^1^	1.0097 × 10^2^	1.0477 × 10^3^	2.2038 × 10^2^	2.8964 × 10^2^	7.8915 × 10^2^	2.4123 × 10^2^	1.1484 × 10^2^	3.5590 × 10^1^	2.8051 × 10^2^	4.5637 × 10^1^	1.2188 × 10^3^	**2.8124 × 10^1^**
F5	Ave	7.2049 × 10^2^	7.3790 × 10^2^	7.4569 × 10^2^	6.1784 × 10^2^	7.3782 × 10^2^	7.0869 × 10^2^	8.6730 × 10^2^	7.7604 × 10^2^	**6.0154 × 10^2^**	8.1510 × 10^2^	6.4601 × 10^2^	7.6744 × 10^2^	6.2108 × 10^2^
	Std	4.3342 × 10^1^	6.4048 × 10^1^	2.5104 × 10^1^	**1.9031 × 10^1^**	3.2245 × 10^1^	4.5410 × 10^1^	6.9395 × 10^1^	2.8327 × 10^1^	2.0869 × 10^1^	6.4080 × 10^1^	2.6337 × 10^1^	3.5289 × 10^1^	4.2142 × 10^1^
F6	Ave	6.5239 × 10^2^	6.5077 × 10^2^	6.6221 × 10^2^	6.1422 × 10^2^	6.5641 × 10^2^	6.3672 × 10^2^	6.8168 × 10^2^	6.6897 × 10^2^	6.1796 × 10^2^	6.6932 × 10^2^	6.3632 × 10^2^	6.6006 × 10^2^	**6.0331 × 10^2^**
	Std	8.1292 × 10^0^	1.4075 × 10^1^	4.0767 × 10^0^	6.4845 × 10^0^	5.9624 × 10^0^	1.2750 × 10^1^	1.3042 × 10^1^	6.1090 × 10^0^	6.5918 × 10^0^	1.0255 × 10^1^	8.3474 × 10^0^	6.4914 × 10^0^	**1.9538 × 10^0^**
F7	Ave	1.1665 × 10^3^	1.0221 × 10^3^	1.1029 × 10^3^	9.0844 × 10^2^	1.1680 × 10^3^	1.1381 × 10^3^	1.3391 × 10^3^	1.3022 × 10^3^	9.0661 × 10^2^	1.6660 × 10^3^	9.8292 × 10^2^	1.2783 × 10^3^	**8.8469 × 10^2^**
	Std	8.8013 × 10^1^	9.0896 × 10^1^	8.4570 × 10^1^	5.2571 × 10^1^	6.9596 × 10^1^	1.9308 × 10^2^	8.4324 × 10^1^	6.7336 × 10^1^	4.2513 × 10^1^	2.1112 × 10^2^	**4.0777 × 10^1^**	4.8551 × 10^1^	5.4867 × 10^1^
F8	Ave	9.7952 × 10^2^	1.0390 × 10^3^	9.6198 × 10^2^	9.1081 × 10^2^	9.9813 × 10^2^	9.9519 × 10^2^	1.0990 × 10^3^	9.8852 × 10^2^	**8.9325 × 10^2^**	1.0208 × 10^3^	9.1286 × 10^2^	9.8897 × 10^2^	9.0543 × 10^2^
	Std	3.8497 × 10^1^	4.4955 × 10^1^	2.2319 × 10^1^	3.6435 × 10^1^	3.3529 × 10^1^	4.3383 × 10^1^	5.4314 × 10^1^	2.9766 × 10^1^	**1.7187 × 10^1^**	3.7050 × 10^1^	2.5265 × 10^1^	2.4012 × 10^1^	3.9214 × 10^1^
F9	Ave	5.3469 × 10^3^	7.0166 × 10^3^	4.8764 × 10^3^	3.1896 × 10^3^	7.3587 × 10^3^	7.5092 × 10^3^	1.2018 × 10^4^	8.8961 × 10^3^	2.1378 × 10^3^	7.6298 × 10^3^	2.9577 × 10^3^	5.6532 × 10^3^	**1.2286 × 10^3^**
	Std	7.2395 × 10^2^	2.3938 × 10^3^	4.7887 × 10^2^	1.1663 × 10^3^	1.6303 × 10^3^	2.1557 × 10^3^	3.7691 × 10^3^	1.3088 × 10^3^	8.4548 × 10^2^	1.6570 × 10^3^	7.6012 × 10^2^	6.2404 × 10^2^	**2.3628 × 10^2^**
F10	Ave	5.6004 × 10^3^	6.7595 × 10^3^	5.4490 × 10^3^	5.4433 × 10^3^	6.5256 × 10^3^	5.5989 × 10^3^	7.4210 × 10^3^	6.2796 × 10^3^	**4.1526 × 10^3^**	5.2567 × 10^3^	6.0116 × 10^3^	5.1951 × 10^3^	6.6265 × 10^3^
	Std	**4.7320 × 10^2^**	1.1877 × 10^3^	6.2398 × 10^2^	1.6346 × 10^3^	9.5552 × 10^2^	7.4067 × 10^2^	7.4526 × 10^2^	8.6116 × 10^2^	8.7096 × 10^2^	6.0515 × 10^2^	1.0685 × 10^3^	4.9117 × 10^2^	7.2366 × 10^2^
F11	Ave	1.3017 × 10^3^	2.1142 × 10^3^	7.6012 × 10^3^	2.4128 × 10^3^	4.4967 × 10^3^	6.0486 × 10^3^	9.6729 × 10^3^	1.6541 × 10^3^	1.4109 × 10^3^	1.5986 × 10^3^	1.6080 × 10^3^	2.1181 × 10^3^	**1.2053 × 10^3^**
	Std	6.3316 × 10^1^	1.0071 × 10^3^	1.8189 × 10^3^	1.0560 × 10^3^	1.4509 × 10^3^	5.2142 × 10^3^	3.9801 × 10^3^	2.9116 × 10^2^	1.0492 × 10^2^	3.1754 × 10^2^	2.0091 × 10^2^	6.2753 × 10^2^	**4.5663 × 10^1^**
F12	Ave	1.6768 × 10^7^	1.0347 × 10^8^	2.1314 × 10^9^	1.9220 × 10^8^	2.8825 × 10^8^	1.7649 × 10^8^	5.1360 × 10^8^	1.0228 × 10^8^	5.1413 × 10^6^	2.7706 × 10^7^	4.7010 × 10^7^	1.1757 × 10^9^	**4.4918 × 10^5^**
	Std	1.4144 × 10^7^	1.4393 × 10^8^	8.1083 × 10^8^	3.4000 × 10^8^	1.7454 × 10^8^	3.3104 × 10^8^	3.8557 × 10^8^	6.8919 × 10^7^	4.8309 × 10^6^	2.7486 × 10^7^	3.6406 × 10^7^	1.3592 × 10^9^	**3.6791 × 10^5^**
F13	Ave	1.4689 × 10^5^	8.0571 × 10^6^	2.6773 × 10^8^	2.0298 × 10^6^	1.7441 × 10^7^	5.5157 × 10^6^	1.8915 × 10^7^	1.1860 × 10^6^	5.9091 × 10^4^	1.2018 × 10^6^	9.6759 × 10^4^	3.0690 × 10^7^	**2.8790 × 10^4^**
	Std	1.2410 × 10^5^	1.5978 × 10^7^	4.2785 × 10^8^	8.2253 × 10^6^	2.3368 × 10^7^	1.8062 × 10^7^	2.4888 × 10^7^	8.5291 × 10^5^	8.5793 × 10^4^	4.4348 × 10^6^	5.1849 × 10^4^	5.6460 × 10^7^	**2.9507 × 10^4^**
F14	Ave	9.2830 × 10^5^	3.9751 × 10^5^	1.2953 × 10^6^	6.5102 × 10^5^	1.3857 × 10^6^	5.9940 × 10^5^	2.6961 × 10^6^	1.2874 × 10^6^	1.0753 × 10^5^	4.4089 × 10^5^	1.7766 × 10^4^	2.7584 × 10^4^	**1.5552 × 10^3^**
	Std	8.3847 × 10^5^	5.4395 × 10^5^	4.0783 × 10^5^	6.2740 × 10^5^	8.9982 × 10^5^	7.7468 × 10^5^	2.2002 × 10^6^	1.7895 × 10^6^	1.1434 × 10^5^	5.1228 × 10^5^	2.1559 × 10^4^	4.6690 × 10^4^	**2.4207 × 10^1^**
F15	Ave	5.2514 × 10^4^	9.1910 × 10^4^	1.8577 × 10^4^	4.0665 × 10^5^	1.9937 × 10^5^	5.8709 × 10^4^	1.1397 × 10^7^	1.2738 × 10^5^	1.9655 × 10^4^	2.5026 × 10^4^	4.0899 × 10^4^	5.7298 × 10^4^	**1.0825 × 10^4^**
	Std	3.9148 × 10^4^	1.0683 × 10^5^	**6.9289 × 10^3^**	8.8015 × 10^5^	1.4827 × 10^5^	3.9732 × 10^4^	1.3251 × 10^7^	6.3222 × 10^4^	1.9376 × 10^4^	1.5264 × 10^4^	3.6586 × 10^4^	3.5408 × 10^4^	1.3809 × 10^4^
F16	Ave	3.2319 × 10^3^	3.3686 × 10^3^	4.0326 × 10^3^	2.6488 × 10^3^	3.4482 × 10^3^	3.1161 × 10^3^	4.5655 × 10^3^	3.7911 × 10^3^	2.6430 × 10^3^	3.2071 × 10^3^	2.8554 × 10^3^	3.0554 × 10^3^	**2.5103 × 10^3^**
	Std	4.5654 × 10^2^	3.9793 × 10^2^	5.8436 × 10^2^	2.7830 × 10^2^	4.3770 × 10^2^	3.3292 × 10^2^	6.6226 × 10^2^	6.1775 × 10^2^	2.4775 × 10^2^	4.3387 × 10^2^	3.3063 × 10^2^	3.4902 × 10^2^	**1.8812 × 10^2^**
F17	Ave	2.5264 × 10^3^	2.5907 × 10^3^	2.9287 × 10^3^	2.1455 × 10^3^	2.4707 × 10^3^	2.5020 × 10^3^	2.7215 × 10^3^	2.7538 × 10^3^	2.1767 × 10^3^	2.6091 × 10^3^	2.1983 × 10^3^	2.2700 × 10^3^	**1.9780 × 10^3^**
	Std	2.7598 × 10^2^	2.9703 × 10^2^	2.3902 × 10^2^	2.2834 × 10^2^	2.6143 × 10^2^	3.0168 × 10^2^	2.5980 × 10^2^	3.3275 × 10^2^	1.9520 × 10^2^	2.7604 × 10^2^	2.1628 × 10^2^	2.0053 × 10^2^	**1.3373 × 10^2^**
F18	Ave	2.0887 × 10^6^	2.9377 × 10^6^	3.1868 × 10^6^	2.1307 × 10^6^	6.6934 × 10^6^	2.7324 × 10^6^	1.9699 × 10^7^	3.7348 × 10^6^	1.2892 × 10^6^	8.3324 × 10^5^	3.3990 × 10^5^	2.5476 × 10^5^	**7.3186 × 10^3^**
	Std	2.7834 × 10^6^	4.1630 × 10^6^	2.4406 × 10^6^	2.8686 × 10^6^	6.1354 × 10^6^	2.8792 × 10^6^	1.9260 × 10^7^	3.9926 × 10^6^	1.3309 × 10^6^	7.6888 × 10^5^	5.4988 × 10^5^	2.7370 × 10^5^	**5.2978 × 10^3^**
F19	Ave	1.5127 × 10^5^	2.3396 × 10^6^	8.6014 × 10^5^	2.9280 × 10^6^	3.8539 × 10^6^	3.9575 × 10^6^	2.4974 × 10^7^	1.6332 × 10^6^	1.7309 × 10^4^	2.1678 × 10^4^	1.0846 × 10^6^	2.0501 × 10^6^	**9.3845 × 10^3^**
	Std	2.4813 × 10^5^	6.6632 × 10^6^	7.7528 × 10^5^	7.9670 × 10^6^	3.4089 × 10^6^	1.3836 × 10^7^	2.1291 × 10^7^	1.2576 × 10^6^	3.6467 × 10^4^	1.8288 × 10^4^	1.7673 × 10^6^	2.9124 × 10^6^	**1.4221 × 10^4^**
F20	Ave	2.7722 × 10^3^	2.7670 × 10^3^	3.1297 × 10^3^	2.4887 × 10^3^	2.7081 × 10^3^	2.6888 × 10^3^	2.9366 × 10^3^	2.8941 × 10^3^	2.4982 × 10^3^	2.9398 × 10^3^	2.4382 × 10^3^	2.4833 × 10^3^	**2.3538 × 10^3^**
	Std	1.7267 × 10^2^	2.5099 × 10^2^	2.4235 × 10^2^	1.8238 × 10^2^	1.8254 × 10^2^	1.9574 × 10^2^	2.3054 × 10^2^	2.2421 × 10^2^	1.6751 × 10^2^	2.3136 × 10^2^	1.5874 × 10^2^	**1.3340 × 10^2^**	1.6947 × 10^2^
F21	Ave	2.5239 × 10^3^	2.5494 × 10^3^	2.6693 × 10^3^	2.4057 × 10^3^	2.5131 × 10^3^	2.4980 × 10^3^	2.6603 × 10^3^	2.5846 × 10^3^	**2.3936 × 10^3^**	2.6203 × 10^3^	2.4290 × 10^3^	2.5429 × 10^3^	2.4111 × 10^3^
	Std	6.5752 × 10^1^	4.4940 × 10^1^	5.4152 × 10^1^	3.1133 × 10^1^	3.9534 × 10^1^	4.1932 × 10^1^	6.0928 × 10^1^	4.1071 × 10^1^	**1.9637 × 10^1^**	7.4386 × 10^1^	2.5478 × 10^1^	6.0633 × 10^1^	3.6748 × 10^1^
F22	Ave	5.5620 × 10^3^	5.6680 × 10^3^	7.7366 × 10^3^	5.9181 × 10^3^	**3.4168 × 10^3^**	6.8336 × 10^3^	8.5582 × 10^3^	6.8831 × 10^3^	4.2866 × 10^3^	6.7022 × 10^3^	5.4021 × 10^3^	5.8898 × 10^3^	3.4728 × 10^3^
	Std	2.4296 × 10^3^	2.4780 × 10^3^	**4.2864 × 10^2^**	2.0898 × 10^3^	9.1580 × 10^2^	1.0953 × 10^3^	9.1975 × 10^2^	1.9949 × 10^3^	2.1098 × 10^3^	1.3599 × 10^3^	2.4407 × 10^3^	1.8157 × 10^3^	2.1520 × 10^3^
F23	Ave	2.9870 × 10^3^	3.0109 × 10^3^	3.9527 × 10^3^	2.7993 × 10^3^	3.0343 × 10^3^	2.8409 × 10^3^	3.1667 × 10^3^	3.3017 × 10^3^	2.8233 × 10^3^	3.1713 × 10^3^	2.8383 × 10^3^	3.0698 × 10^3^	**2.7468 × 10^3^**
	Std	8.9969 × 10^1^	8.8167 × 10^1^	1.9059 × 10^2^	4.1032 × 10^1^	7.5437 × 10^1^	4.0505 × 10^1^	1.0693 × 10^2^	1.3203 × 10^2^	3.7609 × 10^1^	1.1146 × 10^2^	4.9492 × 10^1^	8.3977 × 10^1^	**2.4742 × 10^1^**
F24	Ave	3.1657 × 10^3^	3.1343 × 10^3^	3.9239 × 10^3^	2.9553 × 10^3^	3.1517 × 10^3^	2.9910 × 10^3^	3.2923 × 10^3^	3.5161 × 10^3^	2.9568 × 10^3^	3.3215 × 10^3^	2.9760 × 10^3^	3.1988 × 10^3^	**2.9208 × 10^3^**
	Std	8.5356 × 10^1^	7.6222 × 10^1^	1.9041 × 10^2^	5.1564 × 10^1^	5.7053 × 10^1^	4.0859 × 10^1^	1.2516 × 10^2^	1.5246 × 10^2^	4.4422 × 10^1^	9.5717 × 10^1^	4.3829 × 10^1^	7.5777 × 10^1^	**3.5144 × 10^1^**
F25	Ave	2.9514 × 10^3^	2.9917 × 10^3^	3.2850 × 10^3^	3.0336 × 10^3^	3.1041 × 10^3^	3.2829 × 10^3^	3.2432 × 10^3^	3.0167 × 10^3^	2.9458 × 10^3^	3.0210 × 10^3^	3.0298 × 10^3^	3.3369 × 10^3^	**2.8941 × 10^3^**
	Std	3.4904 × 10^1^	6.6596 × 10^1^	1.2754 × 10^2^	7.3435 × 10^1^	6.9315 × 10^1^	3.8478 × 10^2^	7.4809 × 10^1^	4.6625 × 10^1^	2.4403 × 10^1^	1.1593 × 10^2^	5.6177 × 10^1^	1.8857 × 10^2^	**1.2947 × 10^1^**
F26	Ave	7.0923 × 10^3^	7.1706 × 10^3^	9.0625 × 10^3^	4.9784 × 10^3^	6.7102 × 10^3^	5.9707 × 10^3^	8.6186 × 10^3^	8.2119 × 10^3^	5.5279 × 10^3^	7.7853 × 10^3^	5.6940 × 10^3^	7.8273 × 10^3^	**4.5394 × 10^3^**
	Std	1.0815 × 10^3^	8.6451 × 10^2^	5.5078 × 10^2^	**4.4860 × 10^2^**	1.1973 × 10^3^	4.5563 × 10^2^	1.3748 × 10^3^	1.3613 × 10^3^	4.5786 × 10^2^	7.5189 × 10^2^	8.3970 × 10^2^	1.2656 × 10^3^	6.6184 × 10^2^
F27	Ave	3.3011 × 10^3^	3.3458 × 10^3^	5.4429 × 10^3^	3.2689 × 10^3^	3.4441 × 10^3^	3.2514 × 10^3^	3.4919 × 10^3^	3.5513 × 10^3^	3.3064 × 10^3^	3.5341 × 10^3^	3.3370 × 10^3^	3.3783 × 10^3^	**3.2405 × 10^3^**
	Std	4.1616 × 10^1^	7.5969 × 10^1^	3.7010 × 10^2^	2.4868 × 10^1^	8.6423 × 10^1^	2.3369 × 10^1^	1.1431 × 10^2^	1.5636 × 10^2^	3.5776 × 10^1^	1.3241 × 10^2^	5.4541 × 10^1^	8.2542 × 10^1^	**1.9041 × 10^1^**
F28	Ave	3.3153 × 10^3^	3.6365 × 10^3^	4.4713 × 10^3^	3.4973 × 10^3^	3.8554 × 10^3^	4.4388 × 10^3^	3.7717 × 10^3^	3.5180 × 10^3^	3.3730 × 10^3^	3.3595 × 10^3^	3.3847 × 10^3^	3.9661 × 10^3^	**3.2366 × 10^3^**
	Std	2.8676 × 10^1^	6.9725 × 10^2^	3.7812 × 10^2^	1.6861 × 10^2^	2.0978 × 10^2^	9.2356 × 10^2^	1.9398 × 10^2^	9.6200 × 10^1^	5.6609 × 10^1^	9.3577 × 10^1^	5.9363 × 10^1^	4.0673 × 10^2^	**2.3640 × 10^1^**
F29	Ave	4.5066 × 10^3^	4.6756 × 10^3^	6.3614 × 10^3^	3.9002 × 10^3^	4.7828 × 10^3^	4.1735 × 10^3^	5.5079 × 10^3^	4.8888 × 10^3^	4.0875 × 10^3^	4.5489 × 10^3^	4.2586 × 10^3^	4.6401 × 10^3^	**3.7387 × 10^3^**
	Std	3.0359 × 10^2^	4.7679 × 10^2^	5.1043 × 10^2^	**1.8033 × 10^2^**	2.9762 × 10^2^	2.7585 × 10^2^	7.2088 × 10^2^	3.8721 × 10^2^	2.7289 × 10^2^	3.5968 × 10^2^	2.6168 × 10^2^	3.4279 × 10^2^	2.2720 × 10^2^
F30	Ave	1.6713 × 10^6^	3.2149 × 10^6^	2.4815 × 10^7^	1.1692 × 10^7^	3.6493 × 10^7^	8.9546 × 10^5^	5.0018 × 10^7^	1.0020 × 10^7^	2.1354 × 10^5^	1.4478 × 10^6^	6.1836 × 10^6^	1.1951 × 10^7^	**3.1610 × 10^4^**
	Std	1.1728 × 10^6^	5.2571 × 10^6^	4.0128 × 10^7^	1.1258 × 10^7^	4.1167 × 10^7^	1.8496 × 10^6^	4.5906 × 10^7^	8.6303 × 10^6^	1.7011 × 10^5^	1.3452 × 10^6^	5.7901 × 10^6^	9.4255 × 10^6^	**4.0259 × 10^4^**
Mean		5.50	7.43	10.73	4.90	9.17	7.03	11.93	9.43	2.77	7.87	4.77	7.90	**1.57**
Friedman		5	7	12	4	10	6	13	11	2	8	3	9	**1**

**Table 3:** Experimental results on CEC-2017 test functions (Dim = 50).

ID		AVOA	DBO	GSA	GWO	AO	MFO	WOA	HHO	SO	CPSOGSA	CSA	POA	MIPOA
F1	Ave	2.9488 × 10^8^	9.4080 × 10^9^	5.8525 × 10^10^	1.1238 × 10^10^	2.1174 × 10^10^	3.6740 × 10^10^	2.1849 × 10^10^	5.3502 × 10^9^	5.6369 × 10^8^	9.8981 × 10^9^	4.9085 × 10^9^	4.7961 × 10^10^	**1.9075 × 10^7^**
	Std	4.1905 × 10^8^	2.1055 × 10^10^	8.5037 × 10^9^	4.5354 × 10^9^	4.2105 × 10^9^	1.2578 × 10^10^	3.8991 × 10^9^	2.2065 × 10^9^	2.0998 × 10^8^	4.8078 × 10^9^	1.7571 × 10^9^	1.0061 × 10^10^	**1.0805 × 10^7^**
F2	Ave	5.2902 × 10^52^	3.0335 × 10^68^	1.9988 × 10^84^	6.8962 × 10^53^	3.7373 × 10^70^	1.1951 × 10^77^	3.2775 × 10^79^	9.1011 × 10^67^	5.6281 × 10^55^	1.2402 × 10^73^	1.9635 × 10^60^	1.0619 × 10^63^	**5.5636 × 10^45^**
	Std	1.7013 × 10^53^	1.6613 × 10^69^	7.6309 × 10^84^	1.9680 × 10^54^	1.7965 × 10^71^	6.5456 × 10^77^	1.6477 × 10^80^	4.9842 × 10^68^	2.3272 × 10^56^	6.7922 × 10^73^	9.8052 × 10^60^	3.3317 × 10^63^	**1.4425 × 10^46^**
F3	Ave	1.8919 × 10^5^	2.7656 × 10^5^	1.9488 × 10^5^	1.8156 × 10^5^	3.0632 × 10^5^	4.3045 × 10^5^	2.8589 × 10^5^	1.7202 × 10^5^	1.7097 × 10^5^	3.6711 × 10^5^	1.7478 × 10^5^	1.1233 × 10^5^	**7.9047 × 10^4^**
	Std	3.7198 × 10^4^	8.2206 × 10^4^	**1.2795 × 10^4^**	3.5140 × 10^4^	7.0888 × 10^4^	9.3402 × 10^4^	8.2103 × 10^4^	2.2956 × 10^4^	1.6375 × 10^4^	8.6877 × 10^4^	3.2323 × 10^4^	1.5630 × 10^4^	1.6318 × 10^4^
F4	Ave	7.9972 × 10^2^	1.2816 × 10^3^	1.5654 × 10^4^	1.7345 × 10^3^	4.2896 × 10^3^	3.8929 × 10^3^	5.1159 × 10^3^	1.8554 × 10^3^	8.6837 × 10^2^	3.0884 × 10^3^	1.4543 × 10^3^	8.7806 × 10^3^	**6.1701 × 10^2^**
	Std	6.8260 × 10^1^	2.4237 × 10^2^	2.3240 × 10^3^	7.4702 × 10^2^	1.0757 × 10^3^	1.8051 × 10^3^	1.5429 × 10^3^	3.7131 × 10^2^	1.1678 × 10^2^	1.3211 × 10^3^	2.9461 × 10^2^	3.2381 × 10^3^	**4.9459 × 10^1^**
F5	Ave	8.6339 × 10^2^	9.5158 × 10^2^	8.5549 × 10^2^	7.5049 × 10^2^	9.5613 × 10^2^	1.0049 × 10^3^	1.1374 × 10^3^	9.2720 × 10^2^	**7.2473 × 10^2^**	1.0391 × 10^3^	8.3029 × 10^2^	9.3402 × 10^2^	8.5312 × 10^2^
	Std	3.6645 × 10^1^	1.0630 × 10^2^	3.0082 × 10^1^	3.4811 × 10^1^	4.6990 × 10^1^	7.8366 × 10^1^	9.9959 × 10^1^	**2.8955 × 10^1^**	3.7690 × 10^1^	7.2075 × 10^1^	5.1177 × 10^1^	3.2300 × 10^1^	5.5097 × 10^1^
F6	Ave	6.6139 × 10^2^	6.6989 × 10^2^	6.6796 × 10^2^	6.2677 × 10^2^	6.7490 × 10^2^	6.5927 × 10^2^	6.9638 × 10^2^	6.7881 × 10^2^	6.2991 × 10^2^	6.7750 × 10^2^	6.5141 × 10^2^	6.7277 × 10^2^	**6.1858 × 10^2^**
	Std	6.7602 × 10^0^	1.2371 × 10^1^	**4.3850 × 10^0^**	5.9559 × 10^0^	6.9669 × 10^0^	1.0242 × 10^1^	1.0367 × 10^1^	4.8184 × 10^0^	6.8037 × 10^0^	7.8723 × 10^0^	7.4545 × 10^0^	4.8763 × 10^0^	5.7041 × 10^0^
F7	Ave	1.6519 × 10^3^	1.3877 × 10^3^	1.6426 × 10^3^	1.1682 × 10^3^	1.6843 × 10^3^	1.9944 × 10^3^	1.9250 × 10^3^	1.9366 × 10^3^	1.1925 × 10^3^	2.8421 × 10^3^	1.4487 × 10^3^	1.7881 × 10^3^	**1.1649 × 10^3^**
	Std	1.0896 × 10^2^	1.0469 × 10^2^	1.4455 × 10^2^	9.1817 × 10^1^	1.1312 × 10^2^	3.9865 × 10^2^	1.1419 × 10^2^	**5.8160 × 10^1^**	6.7669 × 10^1^	2.7733 × 10^2^	1.0824 × 10^2^	6.0593 × 10^1^	8.5144 × 10^1^
F8	Ave	1.1556 × 10^3^	1.3441 × 10^3^	1.1798 × 10^3^	1.0665 × 10^3^	1.2817 × 10^3^	1.2915 × 10^3^	1.4126 × 10^3^	1.2323 × 10^3^	**1.0257 × 10^3^**	1.2623 × 10^3^	1.1262 × 10^3^	1.2592 × 10^3^	1.1420 × 10^3^
	Std	4.2960 × 10^1^	9.3757 × 10^1^	3.1160 × 10^1^	3.3294 × 10^1^	4.1198 × 10^1^	9.9951 × 10^1^	6.1294 × 10^1^	3.5641 × 10^1^	3.2201 × 10^1^	7.5343 × 10^1^	5.1284 × 10^1^	**2.9141 × 10^1^**	7.4122 × 10^1^
F9	Ave	1.4385 × 10^4^	2.8105 × 10^4^	1.3259 × 10^4^	1.1974 × 10^4^	2.7467 × 10^4^	1.9996 × 10^4^	3.6869 × 10^4^	3.0994 × 10^4^	7.4608 × 10^3^	2.0580 × 10^4^	1.0322 × 10^4^	1.8820 × 10^4^	**5.1980 × 10^3^**
	Std	1.9930 × 10^3^	6.5856 × 10^3^	**1.2117 × 10^3^**	4.1826 × 10^3^	4.7736 × 10^3^	5.1417 × 10^3^	1.0458 × 10^4^	3.4565 × 10^3^	2.8119 × 10^3^	2.9386 × 10^3^	2.3546 × 10^3^	1.7713 × 10^3^	2.1531 × 10^3^
F10	Ave	8.5399 × 10^3^	1.1447 × 10^4^	8.9688 × 10^3^	9.2901 × 10^3^	1.0876 × 10^4^	8.8719 × 10^3^	1.2958 × 10^4^	9.9956 × 10^3^	9.2331 × 10^3^	**8.2994 × 10^3^**	1.0118 × 10^4^	9.1459 × 10^3^	1.2945 × 10^4^
	Std	1.0602 × 10^3^	2.3969 × 10^3^	8.5049 × 10^2^	2.9036 × 10^3^	9.9065 × 10^2^	1.0871 × 10^3^	1.0118 × 10^3^	7.3108 × 10^2^	2.5339 × 10^3^	9.5288 × 10^2^	1.2169 × 10^3^	8.5722 × 10^2^	**6.3853 × 10^2^**
F11	Ave	1.9064 × 10^3^	4.8591 × 10^3^	2.6014 × 10^4^	8.6195 × 10^3^	7.4590 × 10^3^	1.7793 × 10^4^	8.8642 × 10^3^	2.9668 × 10^3^	3.0027 × 10^3^	1.0658 × 10^4^	7.6358 × 10^3^	6.5115 × 10^3^	**1.5271 × 10^3^**
	Std	4.9749 × 10^2^	3.8733 × 10^3^	2.5387 × 10^3^	3.3053 × 10^3^	1.3234 × 10^3^	1.3330 × 10^4^	2.7252 × 10^3^	7.5452 × 10^2^	9.1004 × 10^2^	6.0534 × 10^3^	2.8034 × 10^3^	2.3661 × 10^3^	**8.5435 × 10^1^**
F12	Ave	1.2816 × 10^8^	8.8557 × 10^8^	4.2222 × 10^10^	1.5256 × 10^9^	6.0273 × 10^9^	6.1298 × 10^9^	4.7445 × 10^9^	1.0448 × 10^9^	6.6404 × 10^7^	1.1879 × 10^9^	4.0195 × 10^8^	1.5201 × 10^10^	**1.4908 × 10^7^**
	Std	1.0248 × 10^8^	5.9534 × 10^8^	7.6290 × 10^9^	1.2612 × 10^9^	2.5178 × 10^9^	5.5883 × 10^9^	2.2008 × 10^9^	6.2141 × 10^8^	3.8887 × 10^7^	8.3149 × 10^8^	2.1266 × 10^8^	7.6058 × 10^9^	**1.0948 × 10^7^**
F13	Ave	1.9198 × 10^5^	9.1156 × 10^7^	1.8275 × 10^10^	1.9944 × 10^8^	7.1639 × 10^8^	1.3179 × 10^9^	5.8800 × 10^8^	4.3662 × 10^7^	2.8365 × 10^5^	2.2196 × 10^8^	2.6945 × 10^5^	3.3974 × 10^9^	**1.8350 × 10^4^**
	Std	1.4580 × 10^5^	9.4112 × 10^7^	5.0417 × 10^9^	1.9651 × 10^8^	5.9688 × 10^8^	1.7404 × 10^9^	3.9032 × 10^8^	5.8616 × 10^7^	2.7713 × 10^5^	7.4438 × 10^8^	3.4000 × 10^5^	3.7507 × 10^9^	**1.1842 × 10^4^**
F14	Ave	1.9826 × 10^6^	3.1650 × 10^6^	5.3122 × 10^7^	1.5637 × 10^6^	6.8938 × 10^6^	3.8267 × 10^6^	6.5792 × 10^6^	6.4221 × 10^6^	7.1914 × 10^5^	2.2667 × 10^6^	4.7079 × 10^5^	5.2724 × 10^5^	**3.4322 × 10^3^**
	Std	1.6501 × 10^6^	3.2302 × 10^6^	4.1359 × 10^7^	1.6266 × 10^6^	5.4659 × 10^6^	5.7949 × 10^6^	6.4888 × 10^6^	6.1494 × 10^6^	6.9083 × 10^5^	1.7449 × 10^6^	4.7969 × 10^5^	5.4959 × 10^5^	**3.5130 × 10^3^**
F15	Ave	5.5944 × 10^4^	2.8480 × 10^7^	8.0970 × 10^8^	2.7787 × 10^7^	1.4206 × 10^7^	9.5692 × 10^7^	6.6677 × 10^7^	4.4927 × 10^6^	3.1480 × 10^4^	6.5757 × 10^4^	4.9029 × 10^4^	1.1832 × 10^8^	**1.5864 × 10^4^**
	Std	2.8993 × 10^4^	7.0773 × 10^7^	9.7517 × 10^8^	3.5476 × 10^7^	2.3529 × 10^7^	2.4528 × 10^8^	6.1391 × 10^7^	7.8951 × 10^6^	1.4945 × 10^4^	2.6338 × 10^4^	4.0995 × 10^4^	2.6096 × 10^8^	**8.1213 × 10^3^**
F16	Ave	4.0674 × 10^3^	4.8276 × 10^3^	6.4482 × 10^3^	3.4314 × 10^3^	4.8263 × 10^3^	4.4784 × 10^3^	6.2912 × 10^3^	5.0899 × 10^3^	**3.4018 × 10^3^**	4.2645 × 10^3^	3.9077 × 10^3^	4.3444 × 10^3^	3.5890 × 10^3^
	Std	4.7607 × 10^2^	5.3483 × 10^2^	1.0992 × 10^3^	5.9658 × 10^2^	5.8225 × 10^2^	4.7190 × 10^2^	1.0299 × 10^3^	7.8245 × 10^2^	**3.3459 × 10^2^**	5.2859 × 10^2^	4.7618 × 10^2^	6.0484 × 10^2^	5.3574 × 10^2^
F17	Ave	3.8067 × 10^3^	4.2014 × 10^3^	4.1340 × 10^3^	**3.0968 × 10^3^**	3.9689 × 10^3^	4.2594 × 10^3^	4.6607 × 10^3^	4.1763 × 10^3^	3.3019 × 10^3^	3.9328 × 10^3^	3.2665 × 10^3^	3.7905 × 10^3^	3.2781 × 10^3^
	Std	3.8872 × 10^2^	4.7803 × 10^2^	4.6209 × 10^2^	3.2423 × 10^2^	4.3696 × 10^2^	8.0164 × 10^2^	8.9091 × 10^2^	4.3448 × 10^2^	2.6438 × 10^2^	4.6381 × 10^2^	2.9908 × 10^2^	6.7560 × 10^2^	**2.5795 × 10^2^**
F18	Ave	6.5948 × 10^6^	1.3885 × 10^7^	4.2481 × 10^7^	1.1388 × 10^7^	2.8860 × 10^7^	1.3867 × 10^7^	5.7629 × 10^7^	1.0989 × 10^7^	4.7092 × 10^6^	3.3140 × 10^6^	2.2322 × 10^6^	5.4385 × 10^6^	**9.9457 × 10^4^**
	Std	5.3562 × 10^6^	1.2919 × 10^7^	1.6636 × 10^7^	1.4774 × 10^7^	1.9180 × 10^7^	1.6496 × 10^7^	4.6556 × 10^7^	7.2744 × 10^6^	4.6628 × 10^6^	3.6018 × 10^6^	1.9019 × 10^6^	5.1674 × 10^6^	**5.4464 × 10^4^**
F19	Ave	4.1188 × 10^5^	6.3471 × 10^6^	1.1335 × 10^8^	1.3832 × 10^7^	5.6507 × 10^6^	8.1226 × 10^6^	1.6107 × 10^7^	2.6191 × 10^6^	5.8936 × 10^4^	4.2008 × 10^5^	3.4819 × 10^6^	4.2511 × 10^7^	**2.2361 × 10^4^**
	Std	3.2485 × 10^5^	8.4324 × 10^6^	1.7736 × 10^8^	2.4460 × 10^7^	5.7245 × 10^6^	3.2492 × 10^7^	1.1870 × 10^7^	1.9346 × 10^6^	4.8360 × 10^4^	4.5216 × 10^5^	4.7841 × 10^6^	7.7919 × 10^7^	**1.7752 × 10^4^**
F20	Ave	3.5788 × 10^3^	3.7859 × 10^3^	3.8316 × 10^3^	3.1774 × 10^3^	3.5167 × 10^3^	3.6889 × 10^3^	3.8909 × 10^3^	3.5784 × 10^3^	3.1563 × 10^3^	3.5304 × 10^3^	3.1398 × 10^3^	**3.1355 × 10^3^**	3.3764 × 10^3^
	Std	3.1675 × 10^2^	4.5224 × 10^2^	3.7715 × 10^2^	4.5326 × 10^2^	2.5740 × 10^2^	4.0955 × 10^2^	3.7242 × 10^2^	3.1435 × 10^2^	3.6805 × 10^2^	3.7946 × 10^2^	3.0056 × 10^2^	2.7130 × 10^2^	**2.0888 × 10^2^**
F21	Ave	2.8104 × 10^3^	2.8860 × 10^3^	2.9720 × 10^3^	2.5695 × 10^3^	2.8399 × 10^3^	2.7403 × 10^3^	3.0665 × 10^3^	2.9333 × 10^3^	**2.5163 × 10^3^**	2.9276 × 10^3^	2.6263 × 10^3^	2.8327 × 10^3^	2.6234 × 10^3^
	Std	1.0630 × 10^2^	7.8216 × 10^1^	6.8312 × 10^1^	7.0242 × 10^1^	7.5107 × 10^1^	6.6141 × 10^1^	1.0763 × 10^2^	8.8300 × 10^1^	**2.9639 × 10^1^**	1.1050 × 10^2^	5.1337 × 10^1^	7.2365 × 10^1^	6.0536 × 10^1^
F22	Ave	1.0619 × 10^4^	1.3114 × 10^4^	1.2097 × 10^4^	1.0455 × 10^4^	1.3063 × 10^4^	**1.0444 × 10^4^**	1.4707 × 10^4^	1.2627 × 10^4^	1.1161 × 10^4^	1.0684 × 10^4^	1.1403 × 10^4^	1.1430 × 10^4^	1.4505 × 10^4^
	Std	8.6504 × 10^2^	1.9851 × 10^3^	**6.9025 × 10^2^**	2.1537 × 10^3^	1.2303 × 10^3^	1.0495 × 10^3^	1.1191 × 10^3^	1.0625 × 10^3^	2.5282 × 10^3^	9.1124 × 10^2^	1.3756 × 10^3^	7.5457 × 10^2^	7.2641 × 10^2^
F23	Ave	3.4346 × 10^3^	3.5165 × 10^3^	4.9777 × 10^3^	3.0441 × 10^3^	3.6680 × 10^3^	3.2082 × 10^3^	3.8320 × 10^3^	4.1264 × 10^3^	3.1209 × 10^3^	3.6138 × 10^3^	3.2126 × 10^3^	3.5988 × 10^3^	**2.9817 × 10^3^**
	Std	1.6022 × 10^2^	1.2222 × 10^2^	3.0108 × 10^2^	7.0946 × 10^1^	1.8497 × 10^2^	8.7919 × 10^1^	2.1537 × 10^2^	2.3516 × 10^2^	**6.1439 × 10^1^**	1.4542 × 10^2^	8.4613 × 10^1^	1.4183 × 10^2^	7.0852 × 10^1^
F24	Ave	3.6269 × 10^3^	3.7577 × 10^3^	5.3076 × 10^3^	3.2543 × 10^3^	3.6988 × 10^3^	3.2442 × 10^3^	3.8877 × 10^3^	4.4272 × 10^3^	3.2251 × 10^3^	3.9792 × 10^3^	3.3390 × 10^3^	3.7097 × 10^3^	**3.2050 × 10^3^**
	Std	1.7616 × 10^2^	1.3517 × 10^2^	2.2834 × 10^2^	1.1254 × 10^2^	1.0978 × 10^2^	**5.4378 × 10^1^**	1.8367 × 10^2^	2.7009 × 10^2^	5.7221 × 10^1^	1.6126 × 10^2^	7.9179 × 10^1^	1.7864 × 10^2^	7.4373 × 10^1^
F25	Ave	3.2832 × 10^3^	3.8680 × 10^3^	8.7620 × 10^3^	3.9485 × 10^3^	4.7558 × 10^3^	5.9075 × 10^3^	5.2830 × 10^3^	3.7516 × 10^3^	3.3063 × 10^3^	4.3009 × 10^3^	3.9362 × 10^3^	7.1105 × 10^3^	**3.1264 × 10^3^**
	Std	5.5758 × 10^1^	1.6992 × 10^3^	7.0851 × 10^2^	4.4225 × 10^2^	4.7516 × 10^2^	2.2091 × 10^3^	6.0823 × 10^2^	1.8737 × 10^2^	1.0370 × 10^2^	8.2893 × 10^2^	1.9838 × 10^2^	1.1911 × 10^3^	**3.1627 × 10^1^**
F26	Ave	1.0378 × 10^4^	1.1515 × 10^4^	1.4031 × 10^4^	7.2903 × 10^3^	1.1017 × 10^4^	8.5578 × 10^3^	1.4768 × 10^4^	1.1918 × 10^4^	7.9383 × 10^3^	1.3595 × 10^4^	9.9146 × 10^3^	1.3092 × 10^4^	**6.3281 × 10^3^**
	Std	1.9493 × 10^3^	1.4145 × 10^3^	8.7333 × 10^2^	**7.4850 × 10^2^**	1.7160 × 10^3^	7.8533 × 10^2^	1.4748 × 10^3^	1.1953 × 10^3^	8.1106 × 10^2^	1.6737 × 10^3^	1.2523 × 10^3^	1.3608 × 10^3^	8.5149 × 10^2^
F27	Ave	3.9191 × 10^3^	3.9766 × 10^3^	9.2313 × 10^3^	3.7201 × 10^3^	4.4317 × 10^3^	3.6407 × 10^3^	4.8363 × 10^3^	5.2461 × 10^3^	3.8844 × 10^3^	4.9020 × 10^3^	3.9096 × 10^3^	4.1680 × 10^3^	**3.4936 × 10^3^**
	Std	1.9781 × 10^2^	2.5968 × 10^2^	8.4420 × 10^2^	**1.1529 × 10^2^**	2.9585 × 10^2^	1.2359 × 10^2^	6.3227 × 10^2^	7.2593 × 10^2^	1.3011 × 10^2^	4.2248 × 10^2^	2.3619 × 10^2^	2.5353 × 10^2^	1.2911 × 10^2^
F28	Ave	3.8564 × 10^3^	6.3669 × 10^3^	9.3534 × 10^3^	4.5984 × 10^3^	6.1137 × 10^3^	8.1093 × 10^3^	6.1288 × 10^3^	4.7950 × 10^3^	4.1609 × 10^3^	4.3380 × 10^3^	4.6260 × 10^3^	6.5867 × 10^3^	**3.4016 × 10^3^**
	Std	2.2606 × 10^2^	2.2933 × 10^3^	7.6380 × 10^2^	4.6861 × 10^2^	4.6043 × 10^2^	1.4225 × 10^3^	5.0333 × 10^2^	3.1439 × 10^2^	3.6346 × 10^2^	3.9793 × 10^2^	4.2688 × 10^2^	7.9843 × 10^2^	**4.4139 × 10^1^**
F29	Ave	5.6170 × 10^3^	6.2899 × 10^3^	2.3271 × 10^4^	4.9119 × 10^3^	8.1554 × 10^3^	5.4509 × 10^3^	9.2375 × 10^3^	7.3594 × 10^3^	5.2124 × 10^3^	6.8630 × 10^3^	6.3723 × 10^3^	7.0882 × 10^3^	**4.4137 × 10^3^**
	Std	5.3195 × 10^2^	9.4608 × 10^2^	4.4061 × 10^3^	**3.4812 × 10^2^**	1.1288 × 10^3^	5.1262 × 10^2^	1.6290 × 10^3^	9.0111 × 10^2^	4.0986 × 10^2^	6.9808 × 10^2^	6.5779 × 10^2^	8.9954 × 10^2^	3.5672 × 10^2^
F30	Ave	3.2765 × 10^7^	5.6864 × 10^7^	1.1849 × 10^9^	1.6428 × 10^8^	2.3845 × 10^8^	5.7091 × 10^7^	3.3749 × 10^8^	1.1754 × 10^8^	1.3016 × 10^7^	1.2403 × 10^8^	1.9690 × 10^8^	5.1439 × 10^8^	**1.8100 × 10^6^**
	Std	1.4764 × 10^7^	5.8220 × 10^7^	6.6602 × 10^8^	7.8330 × 10^7^	8.1920 × 10^7^	1.1046 × 10^8^	1.1172 × 10^8^	5.2713 × 10^7^	7.3199 × 10^6^	4.3400 × 10^7^	5.4431 × 10^7^	8.7463 × 10^8^	**8.8633 × 10^5^**
Mean		4.43	7.73	10.90	4.50	9.50	7.53	11.60	8.67	3.03	7.83	4.73	8.17	**2.37**
Friedman		3	7	12	4	11	6	13	10	2	8	5	9	**1**

**Table 4:** Experimental results on CEC-2017 test functions (Dim = 100).

ID		AVOA	DBO	GSA	GWO	AO	MFO	WOA	HHO	SO	CPSOGSA	CSA	POA	MIPOA
F1	Ave	1.3200 × 10^10^	6.1655 × 10^10^	2.1229 × 10^11^	5.3861 × 10^10^	9.2538 × 10^10^	1.5537 × 10^11^	1.0950 × 10^11^	4.9343 × 10^10^	1.5201 × 10^10^	7.0056 × 10^10^	5.8810 × 10^10^	1.4474 × 10^11^	**6.9105 × 10^9^**
	Std	4.0100 × 10^9^	5.1311 × 10^10^	1.3859 × 10^10^	1.3305 × 10^10^	8.0477 × 10^9^	5.4823 × 10^10^	1.2419 × 10^10^	7.2479 × 10^9^	3.7628 × 10^9^	1.7813 × 10^10^	8.2515 × 10^9^	1.8699 × 10^10^	**2.1666 × 10^9^**
F2	Ave	1.0017 × 10^149^	1.9840 × 10^158^	6.9222 × 10^185^	5.5915 × 10^139^	1.1373 × 10^160^	1.3524 × 10^159^	4.6275 × 10^175^	2.6500 × 10^152^	4.0483 × 10^137^	5.9640 × 10^164^	1.5958 × 10^149^	4.7235 × 10^148^	**1.8367 × 10^124^**
	Std	5.4868 × 10^149^	Infinity	Infinity	2.3675 × 10^140^	Infinity	Infinity	Infinity	1.0068 × 10^153^	2.2164 × 10^138^	Infinity	7.5849 × 10^149^	2.4005 × 10^149^	**9.5923 × 10^124^**
F3	Ave	4.3453 × 10^5^	6.5521 × 10^5^	3.7141 × 10^5^	5.5503 × 10^5^	3.5660 × 10^5^	9.9362 × 10^5^	9.4046 × 10^5^	3.6518 × 10^5^	3.6403 × 10^5^	8.3909 × 10^5^	4.5529 × 10^5^	2.9696 × 10^5^	**2.8789 × 10^5^**
	Std	1.4353 × 10^5^	3.0373 × 10^5^	2.9329 × 10^4^	9.5336 × 10^4^	**1.1530 × 10^4^**	1.5519 × 10^5^	1.1391 × 10^5^	1.0165 × 10^5^	3.8138 × 10^4^	9.8862 × 10^4^	6.2750 × 10^4^	2.3700 × 10^4^	2.3859 × 10^4^
F4	Ave	2.5870 × 10^3^	1.8185 × 10^4^	6.7583 × 10^4^	6.1684 × 10^3^	2.0020 × 10^4^	3.0131 × 10^4^	2.0316 × 10^4^	9.1718 × 10^3^	2.8882 × 10^3^	1.6162 × 10^4^	9.2365 × 10^3^	2.6835 × 10^4^	**1.5762 × 10^3^**
	Std	5.9964 × 10^2^	1.4708 × 10^4^	8.5075 × 10^3^	2.1509 × 10^3^	3.3847 × 10^3^	1.2995 × 10^4^	3.7031 × 10^3^	1.9171 × 10^3^	7.0113 × 10^2^	5.5870 × 10^3^	2.4781 × 10^3^	6.6356 × 10^3^	**2.2160 × 10^2^**
F5	Ave	1.3896 × 10^3^	1.6302 × 10^3^	1.5521 × 10^3^	1.2792 × 10^3^	1.7215 × 10^3^	1.9399 × 10^3^	1.9422 × 10^3^	1.6758 × 10^3^	**1.1756 × 10^3^**	1.8026 × 10^3^	1.5267 × 10^3^	1.6317 × 10^3^	1.5547 × 10^3^
	Std	6.0531 × 10^1^	2.0298 × 10^2^	5.7134 × 10^1^	1.3688 × 10^2^	6.5691 × 10^1^	1.5166 × 10^2^	1.3460 × 10^2^	**4.8275 × 10^1^**	6.7121 × 10^1^	1.3714 × 10^2^	7.7835 × 10^1^	5.5439 × 10^1^	8.0938 × 10^1^
F6	Ave	6.6693 × 10^2^	6.7926 × 10^2^	6.7293 × 10^2^	6.4710 × 10^2^	6.8879 × 10^2^	6.8375 × 10^2^	7.0619 × 10^2^	6.9071 × 10^2^	**6.4697 × 10^2^**	6.8360 × 10^2^	6.7341 × 10^2^	6.8255 × 10^2^	6.5382 × 10^2^
	Std	3.7144 × 10^0^	1.1263 × 10^1^	**3.0742 × 10^0^**	6.3614 × 10^0^	4.2346 × 10^0^	6.2814 × 10^0^	8.0778 × 10^0^	3.6361 × 10^0^	3.1531 × 10^0^	6.7167 × 10^0^	4.7684 × 10^0^	3.6285 × 10^0^	7.5647 × 10^0^
F7	Ave	3.2184 × 10^3^	2.8892 × 10^3^	3.3819 × 10^3^	**2.2279 × 10^3^**	3.5281 × 10^3^	5.9474 × 10^3^	3.8204 × 10^3^	3.7813 × 10^3^	2.2392 × 10^3^	6.8729 × 10^3^	3.2860 × 10^3^	3.4957 × 10^3^	2.2444 × 10^3^
	Std	1.2529 × 10^2^	2.9059 × 10^2^	2.0293 × 10^2^	1.6364 × 10^2^	1.4694 × 10^2^	8.3705 × 10^2^	1.5827 × 10^2^	1.1429 × 10^2^	1.1471 × 10^2^	6.7655 × 10^2^	2.3422 × 10^2^	**6.3721 × 10^1^**	9.5461 × 10^1^
F8	Ave	1.8303 × 10^3^	2.1137 × 10^3^	2.0088 × 10^3^	1.5408 × 10^3^	2.1615 × 10^3^	2.2988 × 10^3^	2.4420 × 10^3^	2.1333 × 10^3^	**1.5190 × 10^3^**	2.1299 × 10^3^	1.8941 × 10^3^	2.0874 × 10^3^	1.8287 × 10^3^
	Std	6.3064 × 10^1^	2.3829 × 10^2^	7.3021 × 10^1^	1.2141 × 10^2^	7.9074 × 10^1^	1.8397 × 10^2^	1.5167 × 10^2^	6.9086 × 10^1^	8.1324 × 10^1^	1.4117 × 10^2^	8.9879 × 10^1^	7.1169 × 10^1^	**5.7080 × 10^1^**
F9	Ave	**2.9381 × 10^4^**	7.7805 × 10^4^	3.0556 × 10^4^	4.7864 × 10^4^	6.6663 × 10^4^	6.3942 × 10^4^	7.4262 × 10^4^	6.9219 × 10^4^	2.9739 × 10^4^	4.6478 × 10^4^	3.8715 × 10^4^	4.1254 × 10^4^	4.6995 × 10^4^
	Std	**2.1581 × 10^3^**	9.4088 × 10^3^	2.7913 × 10^3^	1.3046 × 10^4^	5.9301 × 10^3^	1.0523 × 10^4^	1.5238 × 10^4^	4.1167 × 10^3^	6.9303 × 10^3^	5.3953 × 10^3^	5.7431 × 10^3^	3.9788 × 10^3^	1.0320 × 10^4^
F10	Ave	1.8000 × 10^4^	2.7956 × 10^4^	2.1209 × 10^4^	1.9788 × 10^4^	2.5098 × 10^4^	1.9504 × 10^4^	2.9457 × 10^4^	2.4576 × 10^4^	3.1008 × 10^4^	**1.7018 × 10^4^**	2.3978 × 10^4^	2.0524 × 10^4^	2.9229 × 10^4^
	Std	2.0164 × 10^3^	5.2355 × 10^3^	1.3534 × 10^3^	4.4206 × 10^3^	1.6197 × 10^3^	1.5460 × 10^3^	1.8828 × 10^3^	1.7805 × 10^3^	2.1004 × 10^3^	1.1020 × 10^3^	2.2050 × 10^3^	1.1971 × 10^3^	**8.5091 × 10^2^**
F11	Ave	1.0054 × 10^5^	2.4397 × 10^5^	2.1371 × 10^5^	9.1278 × 10^4^	3.8106 × 10^5^	2.0349 × 10^5^	2.6874 × 10^5^	1.5699 × 10^5^	1.4254 × 10^5^	2.2486 × 10^5^	1.2707 × 10^5^	8.4217 × 10^4^	**3.7970 × 10^4^**
	Std	2.8209 × 10^4^	5.3288 × 10^4^	8.7315 × 10^4^	1.7945 × 10^4^	1.0780 × 10^5^	6.7461 × 10^4^	7.2451 × 10^4^	3.1745 × 10^4^	2.3121 × 10^4^	6.2373 × 10^4^	2.7999 × 10^4^	1.4381 × 10^4^	**8.8541 × 10^3^**
F12	Ave	1.0499 × 10^9^	8.1616 × 10^9^	1.4541 × 10^11^	1.1787 × 10^10^	3.4528 × 10^10^	3.8021 × 10^10^	2.9200 × 10^10^	1.1991 × 10^10^	1.7733 × 10^9^	1.7178 × 10^10^	8.0317 × 10^9^	6.8948 × 10^10^	**2.9368 × 10^8^**
	Std	4.3151 × 10^8^	6.3525 × 10^9^	1.4650 × 10^10^	4.8506 × 10^9^	7.2002 × 10^9^	1.2463 × 10^10^	6.9104 × 10^9^	3.8343 × 10^9^	6.3672 × 10^8^	1.0521 × 10^10^	2.1948 × 10^9^	1.9422 × 10^10^	**1.0510 × 10^8^**
F13	Ave	4.9739 × 10^5^	2.5107 × 10^8^	3.2413 × 10^10^	1.2386 × 10^9^	3.5120 × 10^9^	5.2427 × 10^9^	2.9366 × 10^9^	3.2259 × 10^8^	5.5479 × 10^6^	7.1813 × 10^8^	5.2603 × 10^7^	1.5349 × 10^10^	**1.0355 × 10^5^**
	Std	1.5451 × 10^6^	1.5004 × 10^8^	5.0872 × 10^9^	9.0006 × 10^8^	1.4084 × 10^9^	4.8874 × 10^9^	1.2866 × 10^9^	2.0379 × 10^8^	6.8034 × 10^6^	8.1197 × 10^8^	2.7695 × 10^7^	6.1585 × 10^9^	**2.4429 × 10^5^**
F14	Ave	7.7360 × 10^6^	1.9236 × 10^7^	3.1434 × 10^7^	7.6332 × 10^6^	2.1080 × 10^7^	2.1016 × 10^7^	2.5176 × 10^7^	1.0819 × 10^7^	9.6997 × 10^6^	7.2361 × 10^6^	7.0221 × 10^6^	7.3752 × 10^6^	**6.3074 × 10^5^**
	Std	5.1429 × 10^6^	1.1229 × 10^7^	1.9788 × 10^7^	4.0647 × 10^6^	6.2959 × 10^6^	1.7191 × 10^7^	1.5963 × 10^7^	3.6835 × 10^6^	5.6442 × 10^6^	3.6047 × 10^6^	4.1879 × 10^6^	3.4131 × 10^6^	**6.9134 × 10^5^**
F15	Ave	**8.5075 × 10^4^**	4.1747 × 10^7^	1.5839 × 10^10^	3.7842 × 10^8^	5.5872 × 10^8^	1.4109 × 10^9^	5.1438 × 10^8^	1.6015 × 10^7^	3.2252 × 10^5^	4.7016 × 10^7^	5.2247 × 10^5^	5.1615 × 10^9^	1.8993 × 10^5^
	Std	**8.5283 × 10^4^**	9.0195 × 10^7^	3.3447 × 10^9^	6.6785 × 10^8^	3.2893 × 10^8^	1.3204 × 10^9^	3.6086 × 10^8^	6.7315 × 10^6^	2.3728 × 10^5^	1.4453 × 10^8^	6.1533 × 10^5^	2.6151 × 10^9^	9.8310 × 10^5^
F16	Ave	7.9313 × 10^3^	9.2297 × 10^3^	1.7963 × 10^4^	**6.8128 × 10^3^**	1.1534 × 10^4^	8.2758 × 10^3^	1.6261 × 10^4^	1.0266 × 10^4^	7.3669 × 10^3^	8.6018 × 10^3^	9.2442 × 10^3^	1.1418 × 10^4^	8.7343 × 10^3^
	Std	**9.2130 × 10^2^**	1.5270 × 10^3^	1.8072 × 10^3^	1.1754 × 10^3^	1.5002 × 10^3^	1.1562 × 10^3^	2.3416 × 10^3^	1.4311 × 10^3^	1.7047 × 10^3^	1.0451 × 10^3^	9.9837 × 10^2^	1.7179 × 10^3^	1.1289 × 10^3^
F17	Ave	6.5811 × 10^3^	9.4580 × 10^3^	5.1982 × 10^6^	**5.3805 × 10^3^**	2.6516 × 10^4^	9.8989 × 10^3^	2.6140 × 10^4^	7.8809 × 10^3^	5.7176 × 10^3^	7.0366 × 10^3^	6.4042 × 10^3^	4.2838 × 10^4^	6.4621 × 10^3^
	Std	7.4787 × 10^2^	1.6078 × 10^3^	3.9192 × 10^6^	6.2052 × 10^2^	1.7765 × 10^4^	3.7860 × 10^3^	1.8504 × 10^4^	1.4916 × 10^3^	**5.0660 × 10^2^**	1.1045 × 10^3^	6.2339 × 10^2^	6.1078 × 10^4^	6.8311 × 10^2^
F18	Ave	6.8235 × 10^6^	3.2745 × 10^7^	4.9768 × 10^7^	1.0956 × 10^7^	2.1822 × 10^7^	2.1823 × 10^7^	2.1583 × 10^7^	8.7185 × 10^6^	1.0039 × 10^7^	9.1105 × 10^6^	8.5083 × 10^6^	7.9429 × 10^6^	**1.1131 × 10^6^**
	Std	3.5661 × 10^6^	2.4715 × 10^7^	3.1287 × 10^7^	1.2484 × 10^7^	1.1648 × 10^7^	1.9416 × 10^7^	1.0969 × 10^7^	4.2191 × 10^6^	5.1724 × 10^6^	5.7084 × 10^6^	9.9454 × 10^6^	6.1770 × 10^6^	**6.0917 × 10^5^**
F19	Ave	4.4453 × 10^6^	8.0644 × 10^7^	1.5075 × 10^10^	4.1152 × 10^8^	4.9895 × 10^8^	1.3107 × 10^9^	5.7846 × 10^8^	3.7128 × 10^7^	2.2634 × 10^6^	4.0342 × 10^7^	2.9017 × 10^7^	5.0812 × 10^9^	**1.9765 × 10^4^**
	Std	3.1807 × 10^6^	6.2672 × 10^7^	2.8906 × 10^9^	7.3841 × 10^8^	2.5773 × 10^8^	1.3093 × 10^9^	2.6700 × 10^8^	3.0621 × 10^7^	2.0965 × 10^6^	4.0349 × 10^7^	2.2506 × 10^7^	3.6992 × 10^9^	**5.9227 × 10^4^**
F20	Ave	5.8814 × 10^3^	7.3705 × 10^3^	6.4246 × 10^3^	5.7246 × 10^3^	6.2281 × 10^3^	5.7727 × 10^3^	7.0869 × 10^3^	6.1198 × 10^3^	7.3059 × 10^3^	6.1668 × 10^3^	5.7882 × 10^3^	**5.4629 × 10^3^**	6.4986 × 10^3^
	Std	5.1315 × 10^2^	7.1531 × 10^2^	6.0707 × 10^2^	1.1801 × 10^3^	6.5891 × 10^2^	7.0618 × 10^2^	6.4553 × 10^2^	4.8836 × 10^2^	4.6935 × 10^2^	6.0840 × 10^2^	1.0518 × 10^3^	**3.5643 × 10^2^**	4.1665 × 10^2^
F21	Ave	3.7511 × 10^3^	4.0711 × 10^3^	5.5985 × 10^3^	**3.1015 × 10^3^**	4.1806 × 10^3^	3.7534 × 10^3^	4.4487 × 10^3^	4.3763 × 10^3^	3.1123 × 10^3^	4.2455 × 10^3^	3.4374 × 10^3^	3.9224 × 10^3^	3.3189 × 10^3^
	Std	2.3357 × 10^2^	1.2294 × 10^2^	2.5728 × 10^2^	7.5214 × 10^1^	2.2443 × 10^2^	1.8171 × 10^2^	1.7676 × 10^2^	2.1747 × 10^2^	9.4611 × 10^1^	1.7925 × 10^2^	1.1048 × 10^2^	1.3784 × 10^2^	**6.9626 × 10^1^**
F22	Ave	2.2155 × 10^4^	2.8181 × 10^4^	2.4960 × 10^4^	2.2298 × 10^4^	2.8777 × 10^4^	2.2358 × 10^4^	3.2128 × 10^4^	2.7153 × 10^4^	3.2644 × 10^4^	**2.0252 × 10^4^**	2.7575 × 10^4^	2.4871 × 10^4^	3.2008 × 10^4^
	Std	1.6220 × 10^3^	4.4980 × 10^3^	9.6213 × 10^2^	3.6691 × 10^3^	1.5297 × 10^3^	1.5809 × 10^3^	1.4143 × 10^3^	1.1469 × 10^3^	2.6865 × 10^3^	1.4568 × 10^3^	2.4968 × 10^3^	1.1928 × 10^3^	**8.8935 × 10^2^**
F23	Ave	4.3638 × 10^3^	4.7448 × 10^3^	8.8795 × 10^3^	3.7467 × 10^3^	4.9734 × 10^3^	3.9368 × 10^3^	5.3500 × 10^3^	5.7652 × 10^3^	3.6967 × 10^3^	5.2362 × 10^3^	4.2582 × 10^3^	4.9240 × 10^3^	**3.6046 × 10^3^**
	Std	2.4330 × 10^2^	2.2829 × 10^2^	6.3696 × 10^2^	1.1457 × 10^2^	2.1244 × 10^2^	1.2873 × 10^2^	2.4359 × 10^2^	4.5540 × 10^2^	**9.6990 × 10^1^**	2.0815 × 10^2^	1.6778 × 10^2^	2.6328 × 10^2^	1.0913 × 10^2^
F24	Ave	5.2590 × 10^3^	6.2440 × 10^3^	1.3230 × 10^4^	4.4902 × 10^3^	6.9635 × 10^3^	4.5300 × 10^3^	6.7205 × 10^3^	8.3163 × 10^3^	4.7855 × 10^3^	6.5923 × 10^3^	5.2455 × 10^3^	6.2815 × 10^3^	**4.3069 × 10^3^**
	Std	3.0557 × 10^2^	6.4306 × 10^2^	8.1362 × 10^2^	1.7090 × 10^2^	4.6598 × 10^2^	1.4579 × 10^2^	4.7565 × 10^2^	7.5575 × 10^2^	1.9366 × 10^2^	4.2038 × 10^2^	2.5759 × 10^2^	4.0471 × 10^2^	**1.3167 × 10^2^**
F25	Ave	4.8316 × 10^3^	1.1505 × 10^4^	2.0161 × 10^4^	6.8548 × 10^3^	1.0034 × 10^4^	1.9987 × 10^4^	1.0723 × 10^4^	6.8188 × 10^3^	5.5405 × 10^3^	9.4692 × 10^3^	8.1274 × 10^3^	1.3446 × 10^4^	**4.3240 × 10^3^**
	Std	3.2857 × 10^2^	6.7534 × 10^3^	1.9515 × 10^3^	8.1234 × 10^2^	1.1452 × 10^3^	7.7192 × 10^3^	1.2959 × 10^3^	5.8043 × 10^2^	4.3983 × 10^2^	1.5670 × 10^3^	8.8069 × 10^2^	2.5786 × 10^3^	**2.1478 × 10^2^**
F26	Ave	2.6263 × 10^4^	2.6524 × 10^4^	4.6458 × 10^4^	1.7693 × 10^4^	3.4357 × 10^4^	1.9920 × 10^4^	3.8257 × 10^4^	3.1251 × 10^4^	1.9616 × 10^4^	3.3801 × 10^4^	2.6951 × 10^4^	3.7328 × 10^4^	**1.5828 × 10^4^**
	Std	2.2440 × 10^3^	3.8635 × 10^3^	2.7147 × 10^3^	1.5546 × 10^3^	2.4505 × 10^3^	2.0769 × 10^3^	4.3691 × 10^3^	2.3691 × 10^3^	1.5792 × 10^3^	2.5992 × 10^3^	3.7072 × 10^3^	3.0205 × 10^3^	**1.2757 × 10^3^**
F27	Ave	4.3349 × 10^3^	4.6240 × 10^3^	1.6612 × 10^4^	4.3086 × 10^3^	7.1957 × 10^3^	4.0261 × 10^3^	6.8150 × 10^3^	6.8160 × 10^3^	4.4065 × 10^3^	6.6256 × 10^3^	4.8971 × 10^3^	6.0192 × 10^3^	**3.6151 × 10^3^**
	Std	3.5292 × 10^2^	4.6769 × 10^2^	9.7260 × 10^2^	1.6437 × 10^2^	7.9570 × 10^2^	2.2450 × 10^2^	9.9220 × 10^2^	1.1167 × 10^3^	2.4644 × 10^2^	9.0670 × 10^2^	3.4712 × 10^2^	7.1822 × 10^2^	**1.1644 × 10^2^**
F28	Ave	5.9923 × 10^3^	1.7200 × 10^4^	2.7856 × 10^4^	9.8164 × 10^3^	1.4182 × 10^4^	1.9734 × 10^4^	1.5053 × 10^4^	9.9164 × 10^3^	1.0431 × 10^4^	1.0423 × 10^4^	1.0186 × 10^4^	1.8499 × 10^4^	**4.9444 × 10^3^**
	Std	7.3923 × 10^2^	5.7950 × 10^3^	2.1631 × 10^3^	1.2062 × 10^3^	1.2127 × 10^3^	2.3714 × 10^3^	1.2675 × 10^3^	1.0440 × 10^3^	1.4537 × 10^3^	1.8371 × 10^3^	1.1832 × 10^3^	2.8368 × 10^3^	**4.4709 × 10^2^**
F29	Ave	1.0261 × 10^4^	1.1745 × 10^4^	2.9474 × 10^5^	9.1614 × 10^3^	1.8807 × 10^4^	1.0893 × 10^4^	2.1150 × 10^4^	1.3071 × 10^4^	9.3631 × 10^3^	1.3374 × 10^4^	1.3681 × 10^4^	2.4772 × 10^4^	**8.1815 × 10^3^**
	Std	9.8361 × 10^2^	1.7409 × 10^3^	1.9423 × 10^5^	9.5918 × 10^2^	3.6605 × 10^3^	2.9814 × 10^3^	4.5027 × 10^3^	1.6925 × 10^3^	**8.2832 × 10^2^**	2.1908 × 10^3^	1.9833 × 10^3^	1.7511 × 10^4^	1.1012 × 10^3^
F30	Ave	7.5427 × 10^7^	3.0125 × 10^8^	2.9718 × 10^10^	1.5731 × 10^9^	3.9522 × 10^9^	3.6739 × 10^9^	3.3073 × 10^9^	8.3386 × 10^8^	2.4183 × 10^7^	1.4377 × 10^9^	7.8795 × 10^8^	1.3109 × 10^10^	**6.8659 × 10^5^**
	Std	4.1261 × 10^7^	1.8403 × 10^8^	3.8300 × 10^9^	1.1588 × 10^9^	1.3461 × 10^9^	2.6399 × 10^9^	1.3204 × 10^9^	3.5248 × 10^8^	1.8772 × 10^7^	9.8691 × 10^8^	3.5917 × 10^8^	4.6033 × 10^9^	**6.0340 × 10^5^**
Mean		3.57	7.63	10.73	3.83	9.97	8.17	11.07	7.73	4.30	7.57	5.40	8.13	**2.90**
Friedman		2	7	12	3	11	10	13	8	4	6	5	9	**1**

**Table 5:** Experimental results on CEC-2022 test functions (Dim = 20).

ID		AVOA	DBO	GSA	GWO	AO	MFO	WOA	HHO	SO	CPSOGSA	CSA	POA	MIPOA
F1	Ave	1.9743 × 10^4^	3.7040 × 10^4^	3.1128 × 10^4^	1.5320 × 10^4^	6.2985 × 10^4^	4.7432 × 10^4^	4.0576 × 10^4^	2.6032 × 10^4^	1.8423 × 10^4^	4.1701 × 10^4^	1.5373 × 10^4^	9.4296 × 10^3^	**5.9558 × 10^2^**
	Std	6.9156 × 10^3^	1.5319 × 10^4^	4.8312 × 10^3^	6.3107 × 10^3^	2.0043 × 10^4^	1.7273 × 10^4^	1.7628 × 10^4^	9.4448 × 10^3^	4.9273 × 10^3^	1.5920 × 10^4^	6.0458 × 10^3^	3.6306 × 10^3^	**1.4493 × 10^2^**
F2	Ave	4.8665 × 10^2^	5.1867 × 10^2^	6.4245 × 10^2^	5.2880 × 10^2^	5.8905 × 10^2^	5.3963 × 10^2^	6.4372 × 10^2^	5.6280 × 10^2^	4.6415 × 10^2^	4.8054 × 10^2^	4.9639 × 10^2^	6.2758 × 10^2^	**4.5076 × 10^2^**
	Std	3.9014 × 10^1^	7.3202 × 10^1^	3.9505 × 10^1^	5.1227 × 10^1^	6.7827 × 10^1^	8.4611 × 10^1^	8.6669 × 10^1^	4.3012 × 10^1^	1.9128 × 10^1^	3.4051 × 10^1^	4.2362 × 10^1^	1.1643 × 10^2^	**1.2211 × 10^1^**
F3	Ave	6.4796 × 10^2^	6.3608 × 10^2^	6.6128 × 10^2^	6.0670 × 10^2^	6.4149 × 10^2^	6.1927 × 10^2^	6.7322 × 10^2^	6.6065 × 10^2^	6.0895 × 10^2^	6.6342 × 10^2^	6.2142 × 10^2^	6.5216 × 10^2^	**6.0106 × 10^2^**
	Std	1.0113 × 10^1^	1.3773 × 10^1^	4.1291 × 10^0^	3.7636 × 10^0^	9.3608 × 10^0^	1.1735 × 10^1^	1.7825 × 10^1^	9.8653 × 10^0^	5.5225 × 10^0^	1.2622 × 10^1^	5.5508 × 10^0^	1.0268 × 10^1^	**1.6632 × 10^0^**
F4	Ave	8.9013 × 10^2^	9.0901 × 10^2^	8.7545 × 10^2^	8.5964 × 10^2^	8.8611 × 10^2^	8.9565 × 10^2^	9.3237 × 10^2^	8.9110 × 10^2^	**8.4234 × 10^2^**	9.1128 × 10^2^	8.5532 × 10^2^	8.8483 × 10^2^	8.5471 × 10^2^
	Std	1.5905 × 10^1^	2.8475 × 10^1^	**1.0028 × 10^1^**	3.3781 × 10^1^	1.5547 × 10^1^	2.1758 × 10^1^	3.9353 × 10^1^	1.7643 × 10^1^	1.2986 × 10^1^	2.9791 × 10^1^	1.4114 × 10^1^	1.3026 × 10^1^	1.9780 × 10^1^
F5	Ave	2.3881 × 10^3^	2.0230 × 10^3^	2.0179 × 10^3^	1.3239 × 10^3^	2.6857 × 10^3^	2.8667 × 10^3^	4.1204 × 10^3^	3.0032 × 10^3^	1.3077 × 10^3^	3.2519 × 10^3^	1.3464 × 10^3^	2.1836 × 10^3^	**9.1929 × 10^2^**
	Std	3.5097 × 10^2^	6.7150 × 10^2^	2.2620 × 10^2^	2.8487 × 10^2^	3.9375 × 10^2^	8.5215 × 10^2^	1.4795 × 10^3^	3.1936 × 10^2^	2.6974 × 10^2^	6.3846 × 10^2^	2.2610 × 10^2^	2.6492 × 10^2^	**1.3753 × 10^1^**
F6	Ave	5.3703 × 10^3^	1.3689 × 10^6^	**2.9761 × 10^3^**	1.5393 × 10^6^	4.0700 × 10^5^	5.9060 × 10^6^	5.4454 × 10^6^	2.1487 × 10^5^	8.5441 × 10^3^	8.2087 × 10^3^	3.8881 × 10^3^	1.6092 × 10^6^	5.3856 × 10^3^
	Std	4.9199 × 10^3^	3.5958 × 10^6^	**8.8901 × 10^2^**	3.8649 × 10^6^	3.1340 × 10^5^	1.3341 × 10^7^	5.9085 × 10^6^	1.5240 × 10^5^	5.7790 × 10^3^	6.5980 × 10^3^	2.5991 × 10^3^	4.6748 × 10^6^	6.8396 × 10^3^
F7	Ave	2.1633 × 10^3^	2.1540 × 10^3^	2.3532 × 10^3^	2.0957 × 10^3^	2.1326 × 10^3^	2.1194 × 10^3^	2.2404 × 10^3^	2.1850 × 10^3^	2.0849 × 10^3^	2.2018 × 10^3^	2.0737 × 10^3^	2.1176 × 10^3^	**2.0486 × 10^3^**
	Std	5.4851 × 10^1^	6.3726 × 10^1^	9.4582 × 10^1^	3.2707 × 10^1^	2.6258 × 10^1^	4.5813 × 10^1^	7.5836 × 10^1^	6.0023 × 10^1^	2.7610 × 10^1^	6.2406 × 10^1^	**1.6909 × 10^1^**	3.4188 × 10^1^	2.2005 × 10^1^
F8	Ave	2.2661 × 10^3^	2.3308 × 10^3^	2.5163 × 10^3^	2.2614 × 10^3^	2.2467 × 10^3^	2.2572 × 10^3^	2.3197 × 10^3^	2.2991 × 10^3^	2.2441 × 10^3^	2.3863 × 10^3^	2.2359 × 10^3^	2.2532 × 10^3^	**2.2327 × 10^3^**
	Std	6.1344 × 10^1^	9.6569 × 10^1^	1.0969 × 10^2^	5.1844 × 10^1^	2.4018 × 10^1^	4.1714 × 10^1^	8.6285 × 10^1^	9.3383 × 10^1^	3.6154 × 10^1^	1.3909 × 10^2^	2.1563 × 10^1^	4.2820 × 10^1^	**5.6996 × 10^0^**
F9	Ave	2.4900 × 10^3^	2.5170 × 10^3^	2.7338 × 10^3^	2.5259 × 10^3^	2.6143 × 10^3^	2.5057 × 10^3^	2.5973 × 10^3^	2.5519 × 10^3^	2.4810 × 10^3^	2.4976 × 10^3^	2.5230 × 10^3^	2.5464 × 10^3^	**2.4808 × 10^3^**
	Std	8.1437 × 10^0^	2.7216 × 10^1^	7.7085 × 10^1^	3.1829 × 10^1^	4.9100 × 10^1^	4.1467 × 10^1^	5.3939 × 10^1^	4.0042 × 10^1^	2.0333 × 10^−1^	3.2816 × 10^1^	2.5321 × 10^1^	3.8323 × 10^1^	**3.8046 × 10^−3^**
F10	Ave	3.5991 × 10^3^	3.4209 × 10^3^	5.5186 × 10^3^	3.7981 × 10^3^	3.3083 × 10^3^	4.0603 × 10^3^	5.2546 × 10^3^	4.2522 × 10^3^	**2.8850 × 10^3^**	4.3255 × 10^3^	3.5716 × 10^3^	3.2138 × 10^3^	3.2032 × 10^3^
	Std	7.6443 × 10^2^	1.1973 × 10^3^	5.1475 × 10^2^	8.2774 × 10^2^	1.1749 × 10^3^	9.2667 × 10^2^	1.2851 × 10^3^	7.0083 × 10^2^	**3.8150 × 10^2^**	1.1214 × 10^3^	1.0916 × 10^3^	9.4931 × 10^2^	7.2797 × 10^2^
F11	Ave	**2.8986 × 10^3^**	3.1247 × 10^3^	5.1539 × 10^3^	3.5175 × 10^3^	3.9584 × 10^3^	4.0612 × 10^3^	4.3931 × 10^3^	3.6106 × 10^3^	2.9508 × 10^3^	3.0820 × 10^3^	3.0276 × 10^3^	4.3792 × 10^3^	2.9036 × 10^3^
	Std	1.1758 × 10^2^	1.6101 × 10^2^	8.2614 × 10^2^	3.2366 × 10^2^	5.4425 × 10^2^	8.1659 × 10^2^	1.1580 × 10^3^	7.5041 × 10^2^	**6.6942 × 10^1^**	2.8954 × 10^2^	1.7760 × 10^2^	6.6670 × 10^2^	1.2831 × 10^2^
F12	Ave	2.9957 × 10^3^	3.0353 × 10^3^	4.3917 × 10^3^	2.9813 × 10^3^	3.0511 × 10^3^	**2.9540 × 10^3^**	3.0907 × 10^3^	3.2405 × 10^3^	3.0205 × 10^3^	3.3202 × 10^3^	3.0123 × 10^3^	3.0331 × 10^3^	2.9569 × 10^3^
	Std	4.7298 × 10^1^	6.3750 × 10^1^	3.0243 × 10^2^	2.7349 × 10^1^	4.0003 × 10^1^	**1.1165 × 10^1^**	8.7527 × 10^1^	1.8322 × 10^2^	3.7550 × 10^1^	2.1611 × 10^2^	5.1670 × 10^1^	5.0556 × 10^1^	1.7618 × 10^1^

**Table 6:** Wilcoxon rank-sum test results on the CEC-2017 benchmark functions.

Function	AVOA	DBO	GSA	GWO	AO	MFO	WOA	HHO	SO	CPSOGSA	CPSOGSA	CSA	POA
F1	2.4386 × 10^−9^	3.0199 × 10^−11^	3.0199 × 10^−11^	3.0199 × 10^−11^	3.0199 × 10^−11^	3.0199 × 10^−11^	3.0199 × 10^−11^	3.0199 × 10^−11^	3.0199 × 10^−11^	3.0199 × 10^−11^	3.0199 × 10^−11^	3.0199 × 10^−11^	2.4386 × 10^−9^
F2	2.9215 × 10^−9^	3.0199 × 10^−11^	3.0199 × 10^−11^	3.0199 × 10^−11^	3.0199 × 10^−11^	3.0199 × 10^−11^	3.0199 × 10^−11^	3.0199 × 10^−11^	3.0199 × 10^−11^	3.0199 × 10^−11^	3.0199 × 10^−11^	3.0199 × 10^−11^	2.9215 × 10^−9^
F3	3.0199 × 10^−11^	3.0199 × 10^−11^	3.0199 × 10^−11^	3.0199 × 10^−11^	3.0199 × 10^−11^	3.0199 × 10^−11^	3.0199 × 10^−11^	3.0199 × 10^−11^	3.0199 × 10^−11^	3.0199 × 10^−11^	3.0199 × 10^−11^	3.6897 × 10^−11^	3.0199 × 10^−11^
F4	1.7290 × 10^−6^	5.4941 × 10^−11^	3.0199 × 10^−11^	5.5727 × 10^−10^	3.0199 × 10^−11^	4.9752 × 10^−11^	3.0199 × 10^−11^	3.3384 × 10^−11^	6.7220 × 10^−10^	4.5043 × 10^−11^	4.6159 × 10^−10^	3.0199 × 10^−11^	1.7290 × 10^−6^
F5	9.7555 × 10^−10^	1.5465 × 10^−9^	4.0772 × 10^−11^	**8.4180 × 10^−1^**	3.3384 × 10^−11^	4.3106 × 10^−8^	3.0199 × 10^−11^	3.0199 × 10^−11^	**8.2357 × 10^−2^**	3.0199 × 10^−11^	1.6285 × 10^−2^	3.3384 × 10^−11^	9.7555 × 10^−10^
F6	3.0199 × 10^−11^	3.0199 × 10^−11^	3.0199 × 10^−11^	1.3289 × 10^−10^	3.0199 × 10^−11^	3.0199 × 10^−11^	3.0199 × 10^−11^	3.0199 × 10^−11^	3.0199 × 10^−11^	3.0199 × 10^−11^	3.0199 × 10^−11^	3.0199 × 10^−11^	3.0199 × 10^−11^
F7	3.0199 × 10^−11^	6.5277 × 10^−8^	4.5043 × 10^−11^	**1.5798 × 10^−1^**	3.0199 × 10^−11^	1.2023 × 10^−8^	3.0199 × 10^−11^	3.0199 × 10^−11^	**2.4581 × 10^−1^**	3.0199 × 10^−11^	1.5581 × 10^−8^	3.0199 × 10^−11^	3.0199 × 10^−11^
F8	4.6856 × 10^−8^	1.4643 × 10^−10^	1.3853 × 10^−6^	**3.6322 × 10^−1^**	2.6695 × 10^−9^	7.1186 × 10^−9^	3.0199 × 10^−11^	8.4848 × 10^−9^	**4.7335 × 10^−1^**	3.1589 × 10^−10^	**1.3732 × 10^−1^**	8.4848 × 10^−9^	4.6856 × 10^−8^
F9	3.0199 × 10^−11^	3.0199 × 10^−11^	3.0199 × 10^−11^	5.4941 × 10^−11^	3.0199 × 10^−11^	3.0199 × 10^−11^	3.0199 × 10^−11^	3.0199 × 10^−11^	7.7725 × 10^−9^	3.0199 × 10^−11^	3.0199 × 10^−11^	3.0199 × 10^−11^	3.0199 × 10^−11^
F10	1.2860 × 10^−6^	**8.3026 × 10^−1^**	5.1857 × 10^−7^	4.3531 × 10^−5^	**6.3088 × 10^−1^**	9.5139 × 10^−6^	3.1821 × 10^−4^	**1.2597 × 10^−1^**	5.5727 × 10^−10^	2.0152 × 10^−8^	1.4412 × 10^−2^	8.4848 × 10^−9^	1.2860 × 10^−6^
F11	5.0922 × 10^−8^	3.0199 × 10^−11^	3.0199 × 10^−11^	3.0199 × 10^−11^	3.0199 × 10^−11^	3.6897 × 10^−11^	3.0199 × 10^−11^	3.0199 × 10^−11^	1.2057 × 10^−10^	6.0658 × 10^−11^	5.4941 × 10^−11^	3.0199 × 10^−11^	5.0922 × 10^−8^
F12	8.1527 × 10^−11^	9.9186 × 10^−11^	3.0199 × 10^−11^	3.3384 × 10^−11^	3.0199 × 10^−11^	1.1737 × 10^−9^	3.0199 × 10^−11^	3.0199 × 10^−11^	2.0338 × 10^−9^	3.0199 × 10^−11^	3.0199 × 10^−11^	3.0199 × 10^−11^	8.1527 × 10^−11^
F13	1.0666 × 10^−7^	8.9934 × 10^−11^	1.4294 × 10^−8^	1.2057 × 10^−10^	3.0199 × 10^−11^	2.3897 × 10^−8^	3.0199 × 10^−11^	3.0199 × 10^−11^	7.2884 × 10^−3^	8.6634 × 10^−5^	2.7829 × 10^−7^	4.1997 × 10^−10^	1.0666 × 10^−7^
F14	3.0199 × 10^−11^	3.0199 × 10^−11^	3.0199 × 10^−11^	3.0199 × 10^−11^	3.0199 × 10^−11^	3.0199 × 10^−11^	3.0199 × 10^−11^	3.0199 × 10^−11^	3.0199 × 10^−11^	3.0199 × 10^−11^	3.0199 × 10^−11^	3.0199 × 10^−11^	3.0199 × 10^−11^
F15	2.0283 × 10^−7^	1.1023 × 10^−8^	9.2113 × 10^−5^	1.7769 × 10^−10^	4.0772 × 10^−11^	8.4848 × 10^−9^	3.0199 × 10^−11^	3.0199 × 10^−11^	4.4592 × 10^−4^	2.5974 × 10^−5^	5.5999 × 10^−7^	1.0105 × 10^−8^	2.0283 × 10^−7^
F16	1.1023 × 10^−8^	1.2057 × 10^−10^	3.0199 × 10^−11^	1.3272 × 10^−2^	8.9934 × 10^−11^	1.6947 × 10^−9^	3.0199 × 10^−11^	4.5043 × 10^−11^	1.6955 × 10^−2^	3.4971 × 10^−9^	3.3681 × 10^−5^	3.1967 × 10^−9^	1.1023 × 10^−8^
F17	1.1737 × 10^−9^	1.0702 × 10^−9^	3.0199 × 10^−11^	2.8913 × 10^−3^	1.9568 × 10^−10^	1.1023 × 10^−8^	3.0199 × 10^−11^	4.0772 × 10^−11^	3.3681 × 10^−5^	8.1527 × 10^−11^	7.6588 × 10^−5^	9.8329 × 10^−8^	1.1737 × 10^−9^
F18	3.0199 × 10^−11^	3.0199 × 10^−11^	3.0199 × 10^−11^	3.0199 × 10^−11^	3.0199 × 10^−11^	3.0199 × 10^−11^	3.0199 × 10^−11^	3.0199 × 10^−11^	3.0199 × 10^−11^	3.0199 × 10^−11^	3.0199 × 10^−11^	3.0199 × 10^−11^	3.0199 × 10^−11^
F19	6.7220 × 10^−10^	3.4971 × 10^−9^	6.0658 × 10^−11^	6.0658 × 10^−11^	3.0199 × 10^−11^	6.5261 × 10^−7^	3.0199 × 10^−11^	3.0199 × 10^−11^	3.6709 × 10^−3^	5.6073 × 10^−5^	5.4941 × 10^−11^	3.0199 × 10^−11^	6.7220 × 10^−10^
F20	6.7220 × 10^−10^	3.3520 × 10^−8^	4.0772 × 10^−11^	6.0971 × 10^−3^	1.5581 × 10^−8^	6.5277 × 10^−8^	1.7769 × 10^−10^	2.6099 × 10^−10^	3.5012 × 10^−3^	8.9934 × 10^−11^	**7.7272 × 10^−2^**	3.6709 × 10^−3^	6.7220 × 10^−10^
F21	2.2273 × 10^−9^	4.5043 × 10^−11^	3.0199 × 10^−11^	**4.1191 × 10^−1^**	2.6099 × 10^−10^	5.9673 × 10^−9^	3.0199 × 10^−11^	3.0199 × 10^−11^	3.7782 × 10^−2^	3.6897 × 10^−11^	**5.1877 × 10^−2^**	6.1210 × 10^−10^	2.2273 × 10^−9^
F22	3.1573 × 10^−5^	8.8829 × 10^−6^	1.5581 × 10^−8^	5.2650 × 10^−5^	3.7704 × 10^−4^	2.8790 × 10^−6^	2.8716 × 10^−10^	1.2541 × 10^−7^	3.5638 × 10^−4^	3.5708 × 10^−6^	1.4298 × 10^−5^	8.2919 × 10^−6^	3.1573 × 10^−5^
F23	3.0199 × 10^−11^	3.0199 × 10^−11^	3.0199 × 10^−11^	4.8011 × 10^−7^	3.0199 × 10^−11^	2.3715 × 10^−10^	3.0199 × 10^−11^	3.0199 × 10^−11^	8.1014 × 10^−10^	3.0199 × 10^−11^	8.8910 × 10^−10^	3.0199 × 10^−11^	3.0199 × 10^−11^
F24	3.0199 × 10^−11^	3.0199 × 10^−11^	3.0199 × 10^−11^	8.3146 × 10^−3^	3.0199 × 10^−11^	3.9648 × 10^−8^	3.0199 × 10^−11^	3.0199 × 10^−11^	1.8575 × 10^−3^	3.0199 × 10^−11^	1.1674 × 10^−5^	3.0199 × 10^−11^	3.0199 × 10^−11^
F25	3.4971 × 10^−9^	2.6695 × 10^−9^	3.0199 × 10^−11^	4.0772 × 10^−11^	3.0199 × 10^−11^	9.9186 × 10^−11^	3.0199 × 10^−11^	3.3384 × 10^−11^	3.1589 × 10^−10^	3.0199 × 10^−11^	3.0199 × 10^−11^	3.0199 × 10^−11^	3.4971 × 10^−9^
F26	4.1997 × 10^−10^	3.6897 × 10^−11^	3.0199 × 10^−11^	3.8481 × 10^−3^	2.1947 × 10^−8^	1.4643 × 10^−10^	1.7769 × 10^−10^	1.8567 × 10^−9^	3.3520 × 10^−8^	3.0199 × 10^−11^	3.6459 × 10^−8^	4.6159 × 10^−10^	4.1997 × 10^−10^
F27	4.9980 × 10^−9^	7.3803 × 10^−10^	3.0199 × 10^−11^	7.6588 × 10^−5^	3.0199 × 10^−11^	**1.4945 × 10^−1^**	3.0199 × 10^−11^	3.0199 × 10^−11^	3.4742 × 10^−10^	3.0199 × 10^−11^	1.9568 × 10^−10^	9.9186 × 10^−11^	4.9980 × 10^−9^
F28	2.8716 × 10^−10^	3.3384 × 10^−11^	3.0199 × 10^−11^	3.0199 × 10^−11^	3.0199 × 10^−11^	3.0199 × 10^−11^	3.0199 × 10^−11^	3.0199 × 10^−11^	5.4941 × 10^−11^	1.3289 × 10^−10^	4.0772 × 10^−11^	3.0199 × 10^−11^	2.8716 × 10^−10^
F29	8.1527 × 10^−11^	1.2057 × 10^−10^	3.0199 × 10^−11^	4.8560 × 10^−3^	3.3384 × 10^−11^	4.8011 × 10^−7^	3.0199 × 10^−11^	3.6897 × 10^−11^	6.7362 × 10^−6^	2.3715 × 10^−10^	5.9673 × 10^−9^	3.6897 × 10^−11^	8.1527 × 10^−11^
F30	3.0199 × 10^−11^	1.9568 × 10^−10^	3.0199 × 10^−11^	3.0199 × 10^−11^	3.0199 × 10^−11^	3.3520 × 10^−8^	3.0199 × 10^−11^	3.0199 × 10^−11^	2.3768 × 10^−7^	6.0658 × 10^−11^	3.0199 × 10^−11^	3.0199 × 10^−11^	3.0199 × 10^−11^

**Table 7:** Experimental results of UAV path planning using different algorithms.

Algorithms	Best	Worst	Ave	Std	Rank
AVOA	3.1727 × 10^2^	5.1802 × 10^2^	4.5567 × 10^2^	5.5000 × 10^1^	12
DBO	3.1722 × 10^2^	5.1544 × 10^2^	4.0933 × 10^2^	6.8178 × 10^1^	6
GSA	3.1795 × 10^2^	4.3247 × 10^2^	3.9925 × 10^2^	5.0323 × 10^1^	5
GWO	3.1949 × 10^2^	4.4721 × 10^2^	4.2100 × 10^2^	3.3944 × 10^1^	7
AO	3.2078 × 10^2^	4.5434 × 10^2^	4.2347 × 10^2^	**3.1882 × 10^1^**	9
MFO	3.1722 × 10^2^	5.1541 × 10^2^	3.9273 × 10^2^	5.5584 × 10^1^	4
WOA	3.2204 × 10^2^	5.2814 × 10^2^	4.4443 × 10^2^	5.6567 × 10^1^	10
HHO	3.1847 × 10^2^	5.3456 × 10^2^	4.4723 × 10^2^	7.1323 × 10^1^	13
SO	3.1722 × 10^2^	4.3158 × 10^2^	3.8297 × 10^2^	5.6013 × 10^1^	3
CPSOGSA	3.1933 × 10^2^	5.3707 × 10^2^	4.3887 × 10^2^	5.6984 × 10^1^	11
CSA	3.1722 × 10^2^	5.1540 × 10^2^	4.3633 × 10^2^	7.1451 × 10^1^	8
POA	3.1722 × 10^2^	4.3095 × 10^2^	3.3601 × 10^2^	4.2678 × 10^1^	2
**MIPOA**	**3.1722 × 10^2^**	**4.3083 × 10^2^**	**3.2860 × 10^2^**	3.4660 × 10^1^	**1**

## Data Availability

All data in this paper are included in the manuscript.
